# Application of the Allen Human Brain Atlas in Alzheimer’s disease and Parkinson’s disease

**DOI:** 10.1186/s40035-026-00566-0

**Published:** 2026-07-23

**Authors:** Yi Xiao, Shichan Wang, Yanbing Hou, Huifang Shang

**Affiliations:** 1 Laboratory of Neurodegenerative Disorders, Department of Neurology, Rare Disease Center, National Clinical Research Center for Geriatric, West China Hospital, Sichuan University, Chengdu, 610041 Sichuan China; 2 Department of Geriatrics, West China Hospital, Sichuan University, Chengdu, 610041 Sichuan China

**Keywords:** Neuroimage, Alzheimer’s disease, Parkinson’s disease, Allen human brain atlas, Imaging transcriptomics

## Abstract

Alzheimer’s disease (AD) and Parkinson’s disease (PD) are the two most common age-related neurodegenerative disorders. Allen Human Brain Atlas (AHBA) provides high-resolution transcriptomic data across 102 brain regions with multi-site sampling from healthy controls, promoting the use of brain-wide transcriptomic data for imaging transcriptomics and cross-modal model construction. Increasingly, researchers are utilizing brain-wide transcriptomic datasets to investigate the transcriptome correlates of the neuroimage phenotypes in AD and PD. Leveraging the AHBA, researchers have analyzed the transcriptomic correlations of regional susceptibility to Aβ deposition, tau deposition, α-synuclein propagation, and disease-related multiple-dominal neuroimage phenotypes. These studies revealed that transcriptomic pathways related to metabolism, immunity, neurotransmission, and synaptic function play critical roles in the neuroimage phenotype of AD and PD. By incorporating transcriptomic data modeling, subsequent analyses further confirmed that transcriptomic differences provide the molecular basis for the varying susceptibility observed across brain regions. The analytical approaches of imaging transcriptomics, multimodal data integration strategies, and model construction methods used in AD and PD provide a novel perspective for exploration and can be extended to other neurodegenerative diseases. Future research is expected to utilize brain-wide transcriptomic data to uncover the gene expression mechanisms driving neurodegenerative disease phenotypes.

**Supplementary Information:**

The online version contains supplementary material available at 10.1186/s40035-026-00566-0.

## Background

With advances in transcriptomic sequencing technologies, it is now possible to measure gene expression across the entire human brain, exploring the spatial and molecular characteristics of gene expression [1]. The Allen Human Brain Atlas (AHBA) is a high-resolution transcriptional atlas of the entire normal adult human brain. It provides quantitative transcriptome coverage for approximately 20,000 genes, generated using DNA microarray technology. The atlas covers the entire brain through 102–103 spatially distinct regions sampled from six postmortem adult brains (five males and one female) [2]. While other studies have also studied the adult brain transcriptome, the coverage of brain regions is much smaller than AHBA [3]. AHBA remains one of the most important high-resolution transcriptional profiles of the entire adult brain. Since AHBA is an open-access database, it provides valuable support for emerging fields such as imaging transcriptomics and computational neuroscience. The brain is a highly interconnected organ, with different regions constantly influencing and interacting with one another. Compared to datasets with limited sampling scope and sample size, brain-wide transcriptomic data offer distinct advantages in spatial resolution and integrative potential. The availability of matched anatomical, histological, and imaging data from a same donor brain facilitates multimodal analyses, bridging molecular profiles with neuroimaging data. In addition, brain-wide transcriptomic data enable researchers to construct brain-wide network models and assess the potential impact of specific genes on the transmission and modulation of pathology.

**Fig. 1:**
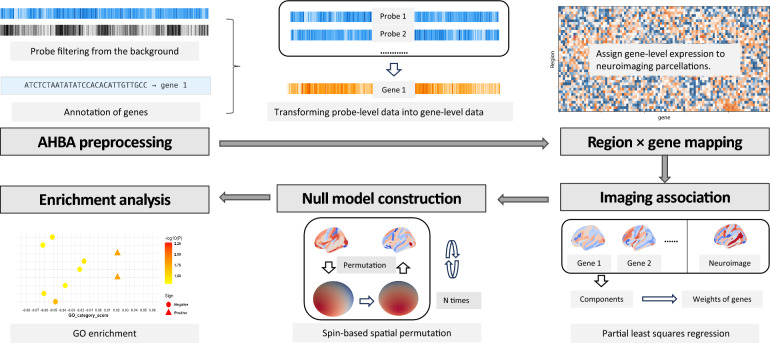
Schematic pipeline for the imaging transcriptomic analyses using AHBA, highlighting the core analytical steps commonly used in imaging transcriptomics

Imaging transcriptomics is an integrative approach that maps gene expression patterns onto imaging features, enabling integration of molecular signatures with structural, functional, and connectivity patterns in the brain as well as biochemical properties [1]. In recent years, many different methods have been used to integrate transcriptome with brain imaging data, although harmony in terms of the workflow of data processing and subsequent analyses was lacking [4]. Recently, a standardized processing pipeline for brain-wide transcriptomic data and a commonly used methodological framework of imaging transcriptomics have been developed (Fig. 1) [5–11].

For example, partial least squares (PLS) regression identifies latent components that explain the covariance between neuroimaging features and spatial gene expression profiles, that is, genes with positive loadings (PLS-positive) are highly expressed in regions showing increased imaging metrics, while genes with negative loadings (PLS-negative) are enriched in areas with decreased imaging metrics. These standardized methods can be referenced from previous literature with detailed and standardized procedures. High-resolution brain-wide maps of cellular abundance for six canonical brain cell types have been derived from AHBA transcriptomic data: neurons, astrocytes, microglia, oligodendrocytes, endothelial cells, and oligodendrocyte precursor cells [12]. These maps enable spatial correlation analyses between cell-type distributions and neuroimaging patterns. Another extension of imaging transcriptomics is the use for mechanistic modeling. AHBA has been leveraged to build in silico models and to generate transcriptome-informed atlases of brain structures. For instance, by assuming that regional transcriptomic profiles influence the spread of pathological proteins, researchers can use the AHBA to predict spatial distribution of these proteins in computational models.

**Fig. 2:**
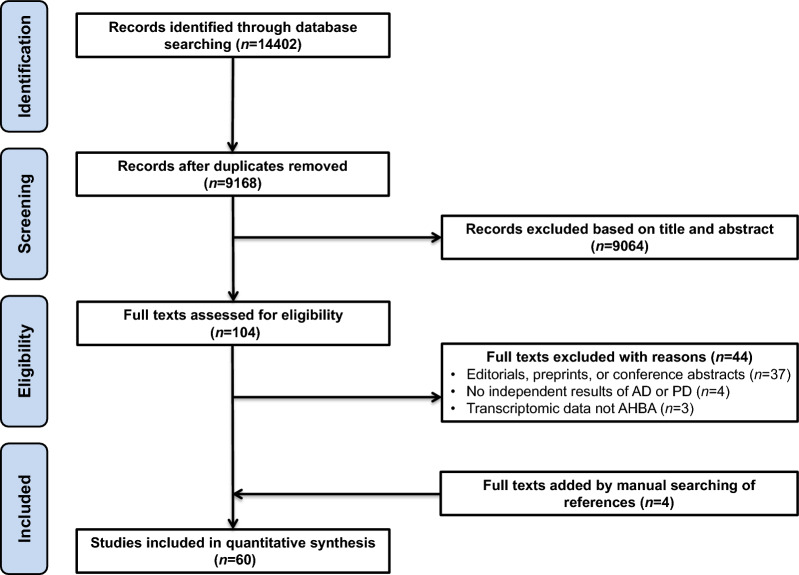
Flow chart of study selection

AD is the most prevalent and burdensome neurodegenerative disorder in terms of patient population and socioeconomic impact, followed closely by PD [13]. AD and PD share several commonalities: their incidence rises with age, and both are primarily characterized by the accumulation of specific misfolded proteins [14, 15]. This review summarizes findings from AD and PD studies utilizing AHBA to conduct imaging transcriptomic analyses or mechanistic modeling, searched from PubMed, Web of Science, Embase, and Scopus databases by the date of July 27, 2025. To ensure comprehensive and targeted literature coverage, we applied a structured search strategy. We included original research articles and quantitative meta-analyses, and excluded narrative reviews that did not involve further data analysis, as well as conference papers and preprints. The complete search string used was: (Alzheimer’s disease OR Parkinson’s disease OR neurodegenerative diseases OR dementia OR Alzheimer OR Parkinson OR neurodegenerat* OR dementia) AND (transcriptome OR RNA-seq OR microarray OR spatial transcriptomics OR imaging transcriptomic OR RNA sequencing OR gene expression atlas) AND (Brain OR Allen Human Brain Atlas). During the screening process, additional relevant articles were identified and included. A total of 14,402 records were retrieved, and 9168 articles remained after removal of duplicates. Following title, abstract, and full-text screening, 60 studies were ultimately included for review (Fig. 2, Tables S1 and S2).

## AD

### Regional susceptibility to and stages of Aβ deposition

Imaging transcriptomics offers a powerful framework to investigate regional susceptibility to Aβ deposition from the genetic perspective. By integrating neuroimaging data with brain-wide gene expression profiles, researchers can uncover the association between transcriptomic features in specific brain regions and Aβ accumulation. This approach enables the identification of spatially patterned transcriptional programs that may underlie the initiation, propagation, and staging of Aβ pathology, providing critical insights into the molecular architecture of AD progression.

#### Characteristics of Aβ-susceptible regions

Combining positron emission tomography (PET) imaging data and AHBA, a previous study found that genes with high expression in Aβ-affected regions are positively enriched in pathways related to synaptic function, lysosomal activity, and axonal structure, while genes with low expression are enriched in pathways related to peptide antigen processing and transmembrane protein complex [16]. Leveraging two large AD datasets, the Alzheimer’s Disease Neuroimaging Initiative (ADNI) and the IMAS (Indiana Memory and Aging Study) cohorts, and based on regional transcriptional data from AHBA, Yu et al. found that genes involved in cell cycle regulation, lipid biosynthesis, protein folding, and membrane transport also show spatial associations with PET Aβ pathology [17]. This partially overlapped with findings from post-mortem AD brains that genes highly expressed in regions with significant Aβ aggregation are enriched in cell cycle, intracellular transport, cellular response to stress, and protein homeostasis pathways [18]. As the impacted region of Aβ pathology was relatively consistent between the studies and the bilateral hemispheres, the different results may have been driven by the different key steps of imaging transcriptomics. The latter two studies [17, 18] used the bilateral cerebral cortex, while the former study [16] only used the left cerebral cortex. Both steps are acceptable, with the use of only left hemisphere data being more common, as in the AHBA only two donors have gene expression data in two hemispheres and the other four have left hemispheric data only [10].

Clustering analyses driven by genetic modules also confirmed the importance of genetic traits in the susceptibility to Aβ accumulation. Based on AHBA gene expression data, a class-balanced ordinal logistic regression model classified brain regions into three Aβ accumulation stages (early, intermediate, and late regions) with 67%–72% accuracy validated by PET, suggesting that molecular identity—not just anatomy—underlies the stage-specific Aβ vulnerability. Enrichment analysis of model-weighted genes revealed that the regions of different stages showed distinct transcriptional profiles, involving genes related to lipid transport, voltage-gated ion channels, and neuropeptide signaling [19]. Together, the brain regions are not randomly affected by Aβ. The transcriptional signatures drive Aβ-vulnerable regions as hubs of high biosynthetic and homeostatic demand, rendering them selectively susceptible to proteotoxic stress in AD.

#### Propagation pathways of Aβ according to PET

Another study identified six distinct cortical propagation pathways of Aβ by PET in cognitively normal older adults, originating primarily from the posterior cingulate cortex and extending to lateral parietal, frontal, and temporal regions [20]. Spatial transcriptomic analysis of 21 previously reported AD-related genes revealed that *CLU* exhibited the strongest spatial correlation with the Aβ propagation map, consistent with a previous study in mice [21]. Further genome-wide exploratory analysis revealed 216 genes spatially correlated with Aβ propagation, with 123 genes correlated with both Aβ and tau propagation. Functional enrichment and interactome analyses revealed that the Aβ-related genes are associated with dendritic structure and transcriptional regulation. In addition, Aβ propagation between neurons was stopped when the dendritic nanotube formation was inhibited in mouse brain [22]. The shared gene set between Aβ and tau propagation was strongly linked to lipid metabolism, with *APOE* occupying a central position in the interactome, suggesting a convergent molecular mechanism underlying tau and Aβ spread across cortical networks [20]. Across independent imaging-transcriptomic cohorts, several genes and biological pathways were repeatedly implicated. *APOE* and lipid metabolism play a key role in the susceptibility to Aβ deposition and transportation of Aβ [19, 20].

#### Functional alterations associated with Aβ deposition

Beyond mapping the spatial and temporal progression of Aβ deposition, recent efforts have sought to understand the functional consequences of Aβ propagation on brain activity using computational neuroscience approaches. Personalized computational models, informed by Aβ-PET and tau-PET data from AD patients, have been developed to simulate blood oxygen level-dependent signals that closely match the real fractional amplitude of low-frequency fluctuation measurements in participants. Imaging-transcriptomic spatial correlation identified 756, 650, and 1987 genes associated with the Aβ-, tau-, and Aβ∙tau-driven activity changes, respectively [23]. The gene set related to the Aβ-driven neuronal activity changes is enriched in inflammation and immune system pathways, as well as in processes of brain development and synaptic function. Pyramidal neurons and endothelial-mural cells are related to Aβ deposition. The gene set associated with interactions of Aβ and tau shows a pronounced enrichment in inflammation, immune response pathways, and microglia [23].

#### Exploring new genes of AD according to Aβ-PET

Imaging transcriptomics offers a new avenue for identifying additional genes that may influence the spatial distribution of molecular pathology in AD [19]. Researchers have identified a group of genes whose spatial expression patterns closely resemble those of PET imaging features of AD, including both known AD-related genes and novel genes potentially associated with AD (e.g., *ASB2, NPY1R, GLRA3, COL23A1, SPRN, CPNE8, TSPAN33*, and *KCNA3*) [24]. Although genome-wide association studies have not identified mutations in these newly discovered genes as being linked to AD, studies of peripheral biomarkers (e.g., *CPNE8* [25], *NPY1R* [26]) and pathological protein propagation mechanisms (e.g., *COL23A1* [27]) suggest that these genes may play a role in the pathogenesis of AD.

### Regional susceptibility to and stages of tau deposition

#### Association between MAPT and tau deposition according to tau-PET

Tau pathology in AD exhibits a stereotyped spatial progression, beginning in the entorhinal cortex and hippocampus and subsequently spreading to neocortical association areas, while primary sensory cortices remain relatively resilient [28]. Emerging imaging transcriptomic evidence suggests that this spatial pattern of tau deposition is closely linked to regional expression of the *MAPT* gene. Across three independent cohorts, higher *MAPT* expression in specific cortical regions was consistently associated with elevated tau-PET signals, and this spatial correlation ranked top 2%–5% among over 18,000 analysed genes. Incorporating *MAPT* expression into connectivity-based models substantially improved the explanatory power for tau accumulation, highlighting its contribution as a molecular determinant of regional vulnerability [29]. Imaging transcriptomic analysis also revealed that the tau propagation map derived from PET imaging data from cognitively normal older adults was most strongly co-localized with *MAPT* gene expression [20]. Another study constructed a tau-PET backbone network from cognitively healthy individuals and identified 577 genes whose expression gradients closely align with the tau propagation trajectory. Among these, *APOE* and *SLC1A2* emerged as central hub genes, both exhibiting high physical interactions with *MAPT* and influencing neurofibrillary tangle protein expression [30]. Together, these findings underscore the pivotal role of *MAPT* and its molecular interactors in shaping the spatial vulnerability landscape for tau pathology in AD.

#### Gene expression signatures mapped onto the tau deposition region/propagation pathway

Gene-to-imaging analyses in two large independent AD cohorts also identified that AD susceptibility genes and gene co-expresion modules are associated with the regional characteristics of tau accumulation. The gene co-expression module characterized by frontoparietal dominance is negatively associated with regional vulnerability to Aβ deposition. In contrast, the frontotemporal-dominant gene module shows positive associations with tau pathology, while the cingulo-sensory-dominant gene module showed negative associations with tau pathology in one cohort. The posterior occipitoparietal-dominant module also showed a significant negative association with tau in one cohort [16].

Genes with high expression in high-tau-deposition regions are enriched in synaptic and postsynaptic signaling, cytosolic ribosome activity, and axoneme assembly, whereas genes related to low expression of tau show negative enrichment in pathways such as extracellular matrix organization and immune-related processes in the two independent tau-PET cohorts of AD patients [16]. Consistently, another AD tau-PET cohort study found that genes with spatial expression patterns significantly associated with regional tau accumulation are strongly involved in synaptic transmission, vesicle trafficking, and neuronal excitability. Protein–protein interaction network analysis further highlighted central hub genes such as *DLG4, SYN1, SYT1, NRXN2*, and *BDNF*—many of which are known to regulate synaptic structure and function [31]. While these results underscore synaptic structure related to regional tau vulnerability, complementary evidence suggested that axonal biology spatially correlates with tau propagation patterns. Data-driven analysis using tau PET imaging data from healthy adult individuals identified 354 genes with cortical expression patterns correlated with tau spread. These tau-related genes are enriched for axon and microtubule organization functions, suggesting that regional vulnerability to tau accumulation may be genetically mediated through axonal structural pathways [20].

By correlating the regional tau burden of the AD cohort and residual tau (the component of tau deposition that could not be explained by the network-based diffusion hypothesis) with the expression of 100 AD risk genes, researchers identified four distinct gene classes: network-aligned vulnerability, network-independent vulnerability, network-aligned resilience, and network-independent resilience [28]. The network-aligned vulnerability genes, such as *MAPT* and *TSPOAP1*, are enriched in biological processes related to neuronal death, stress response, and metabolism, and predominantly expressed in regions like the medial temporal lobe. This is consistent with another study reporting that mitochondrial dysfunction, protein phosphorylation, and cellular stress responses are implicated in tau pathology, as evidenced by the associations of genes like *CDK7, COX17*, and *BACE2* [17]. The network-independent vulnerability genes, including *PRNP* and *JAZF1*, are more strongly associated with residual tau and linked to inflammation and amyloidosis. The network-aligned resilience genes like *BACE1* and *FOXF1* are involved in cellular stress adaptation and neuroprotection, while the network-independent resilience genes such as *SERTAD1* and *MINDY2* are enriched in pathways related to Aβ processing and immune regulation [28]. These findings highlight the differential involvement of various genes related to the tau pathology.

The four studies mentioned above all used ADNI cohort data, yet arrived at two distinct categories of findings—associations of synaptic/axonal processes and metabolic/cellular stress responses with tau pathology in PET imaging —which may stem from differences in how neuroimaging and transcriptomic data were correlated [16, 17, 28, 31]. Spatial autocorrelation effect was considered and controlled using the spatial permutation test in one study [16], while the other three studies did not consider the spatial autocorrelation effect [17, 28, 31]. Therefore, the results of the latter three [17, 28, 31] should be interpreted with caution. Given that both gene expression and tau-PET signals exhibit strong spatial gradients, the reported gene–tau associations in these studies [17, 28, 31] may partially reflect shared spatial structure rather than the molecular signatures of tau vulnerability or propagation.

#### Transcriptomic signatures of tau deposition linked to cognitive decline

To quantify individual-level gene-to-pathology coupling, a previous study introduced pathogenetic scores, which measure the covariance between regional gene expression and pathology burden. Elevated pathogenetic scores were consistently associated with lower cognitive performance measured by Montreal Cognitive Assessment and Mini-Mental State Examination, underscoring the clinical relevance of spatial gene-pathology associations in AD [16]. This study revealed that tau deposition is not only shaped by anatomical connectivity and regional gene expression, but can also reflect individual-level molecular signatures that may predict clinical outcomes.

### Imaging transcriptomic insights into metabolism in AD according to fluorodeoxyglucose-PET (FDG-PET)

In AD, hypometabolism is consistently observed in the posterior cingulate, middle frontal gyrus, angular gyrus, and middle and inferior temporal regions [32]. A spatial transcriptomic study found that *NDUFS4* (which codes for a mitochondrial subunit known to bind oligomeric Aβ) is spatially linked to FDG-PET hypometabolism in AD, suggesting that mitochondrial dysfunction may contribute to energy deficits in affected areas [17]. In addition, of the six brains in the AHBA, one showed a neurofibrillary tangle in the entorhinal cortex and a strong spatial correlation between gene expression and hypometabolism. Robust differential gene expression was found between the normal area and the hypometabolism area. In this brain, genes overexpressed in hypometabolic regions were enriched for endoplasmic reticulum translocation, chronic inflammation, and cell killing, implicating early disruption of protein synthesis and neuroimmune activation [33].

Emerging evidence suggests that type 2 diabetes risk genes may contribute to cerebral metabolic vulnerability in AD through distinct molecular pathways. By correlating FDG-PET standardized uptake values in AD patients with the expression of 46 type 2 diabetes risk genes across 34 cortical regions, researchers identified 15 genes that are significantly associated with brain glucose metabolism in both cognitively healthy individuals and AD patients. These genes are functionally linked to key biological processes, including mitochondrial stability (e.g., *TOMM40, MRPS30*), vascular maintenance and anti-inflammatory signaling (e.g., *PPAP2B, PHACTR1, LPA*), and glucose intolerance and insulin regulation (e.g., *CDKAL1, PAM, DUSP9*) [34].

Insulin resistance (IR) may mechanistically link metabolic dysfunction to core AD pathologies. Researchers have identified strong spatial correlations between AD-related genes (e.g., *MAPT*) and IR signaling components such as IRS-1, GSK-3β, and insulin receptor. These findings offer transcriptomic evidence that supports the connection between IR and AD. Reduced levels of the p-panTyr-IRS-1 (an indicator of effective insulin signaling) in neural-derived plasma exosomes have been observed in AD patients compared to controls, suggesting impaired insulin responsiveness in the AD brain. Importantly, higher p‐panTyr‐IRS‐1 levels in the neural-derived plasma exosomes are associated with less brain atrophy in AD subjects, and normal regional expression of IRS-1 as mapped by the AHBA, is significantly correlated with the volumetric associations. This suggests that these blood-derived biomarkers reflect underlying molecular vulnerability in IRS-1-rich brain regions [35]. In addition, researchers performed a spatial correlation analysis using AHBA and histological maps of Aβ plaques and tau tangles, and revealed strong negative correlations between tau pathology and the expression of several IR-related genes, including *IRS1, AKT1–3, GSK3B,* and *GLUT1*. This suggests that regions with lower expression of these genes are more susceptible to tau aggregation. Conversely, *GLUT4* expression shows a positive correlation with tau density [36].

### Imaging transcriptomic analyses using structural modalities in AD

Structural imaging has revealed gray matter atrophy, white matter connectivity loss, and cortical changes in AD, which correlate with cognitive decline and serve as key biomarkers. Imaging transcriptomics offers molecular insights into these structural disruptions. In this section, we summarize recent structural neuroimaging studies that integrated brain-wide gene expression data. Structural alteration regions show gene expression profiles enriched in synaptic signaling, ion transport, and energy metabolism [12, 37–41].

#### Gene modules associated with structural changes in AD

Gene enrichment analyses consistently revealed that synaptic signaling exhibits a strong spatial correspondence with structural alterations associated with AD. Compared to healthy controls, AD patients showed significantly increased morphometric similarity (MS) in the frontal cortex and parts of the occipital lobe and decreased MS in the temporal and parietal regions [42]. Similarly, morphometric similarity network (MSN) analysis found significantly increased MSN somatomotor network and decreased MSN in the higher-order association cortex in AD patients [43, 44]. These MS alterations were closely linked to cognitive decline. Regions with decreased MS were significantly associated with a set of genes involved in synaptic signaling, neurotransmitter release, and cognition [42]. In addition, synaptic signaling genes were confirmed to be downregulated in the brain tissues of AD [42]. Enrichment analysis further revealed that the MSN-associated PLS-positive genes were involved in transcriptional regulation, whereas MSN-associated PLS-negative genes were enriched in synaptic signaling, oxidoreductase activity, and the neurodevelopmental pathway [44]. Regional Radiomics Similarity Network (R2SN) approach is another framework that quantifies inter-regional morphological similarity. The regional mean connectivity strength (RMCS) derived from R2SN was significantly altered in key regions such as the hippocampus and medial temporal lobe in AD patients, correlating with cognitive decline [40, 45]. PLS regression identified genes that are enriched in modulation of chemical synaptic transmission, regulation of ion transport, synaptic signaling, and brain development are associated with changes in RMCS [40, 45].

By integrating brain-wide microarray data with diffusion tensor imaging, a previous study precisely mapped the expression of AD-related genes within hippocampal fiber pathways. Protein interaction network analysis highlighted key nodes such as GSK3B, CASP3, and GRIA1—proteins closely linked to amyloid precursor protein and implicated in synaptic dysfunction and neurodegeneration [46]. Another study identified eight genes (*ABCA7, SORCS1, SORL1, PILRA, PFDN1, PLXNA4, TRIP4,* and *CD2AP*) with positive associations with gray matter volume change in the clinical AD spectrum, and four genes (*CD33, PLCG2, APOE*, and *ECHDC3*) with negative association [47]. These genes are separately involved in lipid metabolism, synaptic integrity, immune signaling, or protein trafficking. A meta-analysis revealed cortical thinning in the bilateral parahippocampal gyrus, the left posterior cingulate cortex, the orbitofrontal cortex, the sensorimotor areas, and the temporal lobe in AD, and gene sets related to the cortical thinning are enriched for cellular zinc ion homeostasis, ion-gated channel activity, and integral components of the plasma membrane [37].

Brain regions that exhibit enriched expression of synaptic signaling-related genes under normal physiological conditions appear to be particularly vulnerable to AD structural changes. Notably, this correlation precisely aligns with the observed Aβ and tau distribution in imaging-transcriptomic studies, as Aβ and tau preferentially target synaptic structure. Thus, the multimodal evidence supports a model in which baseline synaptic integrity, while essential for cognitive function, paradoxically confers heightened sensitivity to AD-related pathological processes, ultimately driving region-specific atrophy and cognitive decline.

#### Cell types associated with structural changes in AD

In both early- and late-onset AD, tissue loss shows strong positive associations with increased astrocyte and microglial densities [12], where the cell-type atlas was derived from AHBA. Consistently, genes significantly positively associated with decreased MS are enriched in astrocytes, based on cell-type annotations from an independent human RNA-seq dataset GSE73721 [42]. Cell-type mapping further revealed that the genes related to the MSN changes in AD are predominantly expressed in astrocytes and neurons [43], and that the R2SN values are significantly positively correlated with neuronal signatures, whereas those of endothelial cells and oligodendrocytes showed negative correlations [40]. Both of the analyses [40,43] leveraged a common cell-type atlas synthesized from five single-cell studies [48–52] on postmortem cortical samples from human postnatal subjects.

#### Distinct gene modules associated with AD subtypes

AD subtypes display different patterns of gray matter atrophy. Subgroup-specific analysis of genetic associations with gray matter volume revealed cell cycle genes in AD-Memory (AD with predominant memory impairment), protein metabolism genes in AD-Language, and transcriptional regulation genes in AD-Visuospatial. These findings suggest that distinct biological pathways may be involved in the neurobiological differences across AD subgroups [38]. In addition, compared to the Aβ^−^/tau^−^ subjects, the Aβ^+^/tau^+^ subjects exhibited elevated MS in the caudal anterior cingulate and lateral occipital cortex, and reduced MS in frontal, parietal, and visual regions. The MS-increased regions were associated with immune-related genes expressed in astrocytes, microglia, and oligodendrocyte precursors. In contrast, the MS-decreased regions were linked to neuronal genes involved in transmembrane transport and energy metabolism [41]. However, as spatial autocorrelation was not controlled for in this study, the associations may be inflated by shared spatial structure. More spatially robust analyses are needed [41].

### Imaging transcriptomic analyses using functional modalities in AD

In AD, the posterior cingulate cortex/precuneus, parahippocampal gyrus, temporal lobe/medial temporal structures, default mode network (DMN), and sensorimotor/somatomotor networks exhibit convergent structural and functional changes. Structurally, these regions show cortical thinning and radiomics similarity reductions [40, 42–47]; functionally, they display disrupted intrinsic activity, abnormal connectivity, and gradient reorganization [37, 53–56]. Imaging transcriptomic analyses revealed that the functional changes are also spatially correlated with gene expression profiles enriched in pathways of regulation of ion channel activity and synaptic signaling [37, 53–56]. The observation that AD‑related genes show distinct expression patterns during fetal and adolescent stages suggests that the functional network disruptions in AD may be linked to the activation of molecular programs in critical developmental windows [56, 57].

#### Gene modules associated with regional/network functional changes in AD

A meta-analysis revealed decreased intrinsic activity in the bilateral posterior cingulate cortex/precuneus and the right angular gyrus, alongside increased activity in the bilateral parahippocampal gyrus in AD patients. Gene enrichment analysis indicated that the functional abnormalities are associated with genes enriched in the G protein-coupled receptor signaling pathway, ion-gated channel activity, and transmembrane receptor functions [37].

Another meta-analysis identified 46 brain regions within the DMN that exhibit significant connectivity alterations in AD patients. A biclustering approach revealed a subset of 38 genes with distinct co-expression patterns between AD-affected and unaffected DMN regions. Notably, these genes were significantly enriched in pathways related to synaptic signaling and *ERBB* family regulations [53]. AD patients also show reduced average controllability and increased modal controllability in the DMN, and the opposite pattern is observed in the limbic network. These shifts suggest a dysregulation in the brain’s ability to transition between cognitive states. Genes linked to regions with increased network controllability are predominantly involved in ion channel regulation, synaptic signaling, and neuronal excitability. Conversely, genes associated with regions showing reduced controllability are enriched in neurotransmitter receptor activity, synaptic organization, and nervous system development [56].

In addition, the global connectome gradient is disrupted in AD, with primary and secondary gradient scores abnormally elevated in somatomotor networks, and significantly decreased in the DMN region [54, 55]. These gradient abnormalities reflect a reorganization of large-scale brain network architecture and are closely linked to cognitive impairments. Transcriptome-connectome association analysis further identified that the first gradient alterations are spatially linked to gene expression profiles enriched in potassium ion transport and protein-containing complex remodeling [54]. Genes associated with the altered secondary gradients were enriched in neurobiological pathways—such as synaptic signaling, neuronal differentiation, and neurotransmitter transport [55]. Notably, these genes are characterized by dynamic expression patterns during normal human development, with differential regulation observed between prenatal and postnatal stages.

#### Gene expression associated with repetitive transcranial magnetic stimulation (rTMS) response in AD

rTMS-induced neuroplasticity is highly consistent with the previous transcriptional findings associated with network alterations in AD. Weeks of rTMS significantly promoted reconstruction of functional information integration in the multiple roles of 27 regions associated with the brain overlapping system, involving the attentional network, sensorimotor network, default mode network and limbic network, correlating with improvements in episodic memory. Genes spatially correlated with the network alterations were enriched in cell–cell signaling, synaptic transmission, and transsynaptic communication. These genes are predominantly expressed in brain tissue, enriched in immune-related cell types, and showed peak expression during fetal and adolescent developmental stages. Protein–protein interaction analysis identified *RPL11, HSPA4,* and *HTT* as central nodes of the gene-connected network [57].

#### Gene modules associated with white matter functional changes in AD

White matter functional connectivity refers to the synchronized neural activity across white matter tracts, reflecting their role in facilitating long-range communication between gray matter regions. AD-specific impairments of the white matter functional connectivity are localized to the anterior and posterior limbs of the internal capsule, corona radiata, and left tapetum. Imaging transcriptomic analysis revealed that the white matter dysfunctions in AD are associated with gene expression profiles enriched in biological processes such as synapse organization, trans-synaptic signaling, and neuron projection development. These genes are predominantly expressed in neurons and astrocytes [39].

### Imaging transcriptomic analyses using specialized modalities in AD

Excess iron in the brain can disrupt normal cellular processes by generating reactive oxygen species, inducing ferroptosis and accelerating inflammation [58]. Genes highly expressed in regions with high paramagnetic values (regions with significant iron aggregation) in post-mortem AD brains are related to synaptic signaling, regulation of transport, establishment of localization in cell, post‐translational protein modification, cellular response to stress, and cell death [18]. Another in vivo study found that genes related to the distribution of iron accumulation are mainly enriched for protein phosphorylation and metal ion transport. Additionally, these genes are expressed in microglia and glutamatergic neurons [59]. Brain tissue electrical conductivity, a novel metric derived from multi-echo gradient-recalled echo-based B1 phase mapping, reflects the ionic microenvironment and cellular composition of neural tissue. In AD, electrical conductivity is significantly elevated in regions such as the frontal, temporal, and cingulate cortices, correlating with Aβ and tau aggregation as well as cognitive decline. Imaging transcriptomic analyses revealed that the electrical conductivity changes are associated with upregulated expression of genes involved in ion transport and signaling pathways [60].

### Other novel model constructions using brain-wide gene expression atlases in AD

Beyond leveraging the spatial correspondence between brain-wide transcriptomic data and neuroimaging to explain structural and functional alterations in AD, brain-wide transcriptomic data also provide a molecular foundation for constructing novel brain models aimed at understanding and exploring the molecular mechanisms underlying AD. These models hold promise for future extension to other age-related neurodegenerative diseases.

The Gene Expression Multifactorial Causal Model uses differential equations modulated by gene expression to capture how hundreds of genes affect six key biological factors—Aβ, tau, cerebral blood flow, glucose metabolism, neuronal activity, and gray matter density—and their interactions across brain networks. This model quantifies individual-level gene dysregulation patterns by fitting transcriptomic-imaging interaction parameters for each subject, thereby establishing causal pathways from gene activity to cognitive decline. Using this model, researchers have identified 111 genes with stable causal contributions to the macroscopic factor interactions (tau and Aβ burdens, vascular flow, glucose metabolism, functional activity, and atrophy) and associated with the cognitive changes in AD [61]. These genes were enriched in biological pathways related to glucose metabolism [34], inflammation [28], and oxidative stress [18, 28], which were consistent with previous single-modality imaging transcriptomic analyses.

To identify novel AD candidate genes, researchers integrated AHBA data with genome-wide association data and *Drosophila* phenotypes, to find out fly orthologs of genes that cause AD-like traits when disrupted. This integrative approach identified two statistically supported novel AD-related genes: *EPS8*, involved in actin cytoskeleton regulation and synaptic plasticity, and *HSPA2*, a heat shock protein implicated in protein folding and neurodegeneration [62]. Previous studies have shown that deletion of *EPS8* impairs hippocampal synaptic plasticity and leads to cognitive deficits in mice [63]. *HSPA2* is implicated in AD by several pieces of evidence. *HSPA2* is upregulated in AD brain samples, identified as a key driver in transcriptomic and proteomic networks, and experiments in cell models validated that overexpression of *HSPA2* increases Aβ and tau levels [64].

In addition, using a modularity-constrained logistic regression model, Xie et al. identified functionally connected SNPs, genes and proteins related to AD, based on the genotype, gene expression and protein expression data collected from the frontal cortex region in the ROS/MAP cohort. The brain-wide expression profiles of these genes in the AHBA are strongly correlated with the brain activation map associated with visual processing, as well as cognitive functions such as memory and recognition. These results highlight the involvement of the frontal cortex in both structural and functional disruptions in AD [65].

## PD

### Regional susceptibility to Braak stages and α-synuclein spread

PD is a progressive neurodegenerative disorder characterized by the accumulation of misfolded α-synuclein protein aggregates, known as Lewy bodies, and the selective loss of dopaminergic neurons, particularly in the substantia nigra. Despite extensive research, the mechanisms underlying the spatial and temporal progression of PD pathology across the brain remain incompletely understood. Recent advances in transcriptomics, neuroimaging, and computational modeling have enabled more integrative exploration of how transcriptomic characteristics and network converge to shape disease propagation. In particular, the classic Braak staging framework has provided a valuable anatomical basis for mapping the sequential involvement of brain regions, offering a foundation for investigating the regional vulnerability of PD pathology.

#### Transcriptomic features related to Braak stages

The Braak staging describes the caudal-to-rostral progression of Lewy body pathology in PD, delineating six sequential brain regions (R1–R6). Brain-wide transcriptomic data analysis using AHBA data identified 960 Braak stage-related genes whose expression either increases (612 genes) or decreases (348 genes) across these regions. Most of the regional differential expressions have been confirmed in the microarray and RNA-seq data from healthy controls and PD patients [66]. These Braak stage-related genes are enriched for biological processes such as synaptic signaling and neurodevelopment (positive correlation) or vascular morphogenesis and structural organization (negative correlation) [66].

#### Integrative modeling and experimental validation of α‑synuclein spread in PD

The prion-like hypothesis suggests that the spread of misfolded α-synuclein proteins may resemble prion infections and likely occurs along the brain’s structural connections [67]. The network diffusion model conceptualizes disease spread as a diffusion process along the brain’s structural connectivity network. By simulating pathology seeding at each brain region and comparing predicted atrophy patterns with magnetic resonance imaging (MRI)-derived maps from 232 de novo PD patients, Freeze et al. identified the substantia nigra as the most likely origin of disease propagation. Clinically, this region exhibits early and severe dopaminergic neuron degeneration, and abundant Lewy body pathology, and is critically involved in the hallmark motor deficits of PD. This model further predicted early involvement of subcortical regions such as the red nucleus, subthalamic nucleus, and pallidum. These predictions are strongly supported by regional transcriptional profiles: brain regions with high expression of lysosomal and immune-related PD risk genes—such as *GBA, TMEM175, HLA-DQA1*, and *IL1R2*—are more likely to serve as seed regions and show faster predicted arrival times of pathology [68].

Using stepwise functional connectivity analysis seeded from the medulla, another study reconstructed the propagation trajectories of brain connectivity in healthy individuals and compared them with functional disruptions observed in PD patients. Remarkably, these connectivity pathways mirrored the anatomical distribution of α-synuclein pathology seen in postmortem samples, reinforcing the hypothesis of trans-neuronal disease spread. By integrating cortical gene expression data from the AHBA, the study revealed that the spatial expression pattern of *SNCA* highly aligned with the stepwise functional connectivity-derived propagation map. Further enrichment analysis identified a gene set strongly associated with microtubule-related cellular components, suggesting that cytoskeletal architecture may play a critical role in mediating regional vulnerability and facilitating the spread of PD pathology [69].

Zhang et al. developed a susceptible-infected-removed agent-based model integrating the brain structural network and regional gene expression to simulate the spread of neurotoxic proteins. Structural connectivity, derived from diffusion MRI, defines the anatomical pathways along which misfolded α-synuclein proteins can propagate. Functional connectivity, obtained from resting-state functional MRI, modulates the likelihood of propagation along these structural routes by incorporating patterns of neuronal coactivation, thereby refining the dynamics of spread. Regional gene expression profiles of *SNCA* and *GBA* define the local synthesis and clearance rates of α-synuclein in the model. The susceptible-infected-removed model also successfully reproduced the spatial pattern of brain atrophy observed in PD patients and pinpointed the substantia nigra as the most likely epicenter of disease propagation [70]. The enhanced model further demonstrated that incorporating *LRRK2* gene expression—mapped to the probability of protein secretion—significantly improved the fit to empirical atrophy data [71].

To confirm the spatiotemporal dynamics of α-synuclein pathology, Dadgar-Kiani et al. employed a wild-type mouse model in which pre-formed α-synuclein fibrils were stereotaxically injected into various brain regions [72]. Longitudinal brain-wide imaging using tissue clearing and light-sheet microscopy revealed a biphasic pattern of pathology—initial widespread propagation followed by regional decay over 18 months, which was consistent with the spread model revealed by Braak stages [66]. To mechanistically capture these dynamics, the authors constructed a brain-wide computational model with parameters of α-synuclein pathology aggregation, retrograde spreading, and decay. This model was grounded in the mesoscale anatomical connectivity derived from the Allen Mouse Connectivity Atlas and further refined by integrating spatial transcriptomic data from the Allen Brain Atlas from adult mice. The *Lrrk2* and *Gba* genes improved the model’s regional predictions when incorporated into the spreading and the decay parameters, respectively. Moreover, cell-type-specific patterns emerged: genes enriched in neurons were associated with spreading, whereas those expressed in oligodendrocytes, such as *MBP*, correlated with decay. Importantly, gene rankings remained highly consistent across different α-synuclein seeding sites, underscoring a robust and generalizable genetic architecture underlying disease progression [72].

Taken together, these multi-scale investigations—from transcriptomic gradients and network-based diffusion models to integrative simulations and in vivo validations in mice—converge on a unified framework for understanding PD progression. They underscore the critical interplay between structural/functional connectivity and gene expression (*SNCA, GBA, LRRK2*) in shaping the spatiotemporal dynamics of α-synuclein pathology.

### Imaging transcriptomic analyses using structural modalities in PD

Structural alterations represent a core neuroanatomical feature of PD, manifesting across multiple cortical and subcortical regions and evolving dynamically throughout disease progression. Neuroimaging studies have identified region-specific patterns of gray matter atrophy, cortical thinning, morphometric disruptions, and network disruptions in PD patients, with distinct spatial patterns observed at different disease stages and symptom profiles. These structural changes reflect underlying molecular and cellular vulnerabilities, as revealed by integrative spatial imaging transcriptomic analyses. Recent studies have uncovered key biological pathways—ranging from mitochondrial function and synaptic signaling to glial cell activity and neurotransmitter receptor expression—that may drive or modulate structural degeneration in PD.

#### Transcriptomic signatures related to different disease durations of PD

Neuroimaging meta-analysis revealed gray matter atrophy in the bilateral superior temporal gyrus and inferior parietal gyrus, the right angular gyrus, and the left superior frontal gyrus in PD. Analysis of the link between brain transcriptomic data and neuroimaging phenotypes identified genes associated with atrophy, which were predominantly expressed in dopaminergic receptor neurons and enriched in biological processes and molecular pathways including potassium channels, cell–cell communication, neurotransmitter receptors, and transmission across chemical synapses [73].

In the early-stage PD patients from PPMI, gray matter atrophy is most prominent in the occipital lobe, followed by the temporal lobe and basal ganglia [74]. Among 17 PD-related genes analyzed, *LAG3* and *NUCKS1* showed positive correlations with atrophy, while *RAB5A* showed a negative correlation [74]. Oligodendrocyte abundance is most strongly linked to atrophy [12]. In drug naïve PD patients, the MS value decreased in the cingulate, frontal, and temporal cortical areas and increased in the parietal and occipital cortical areas. Genes associated with changes in MS are involved in astrocytes and neurons, and functionally enriched in neuron‐specific biological processes related to trans‐synaptic signaling and nervous system development [75]. PD with longer disease duration (average 5.3 years) has reduced gray matter properties in the temporal, somatomotor, cingulate, and occipital cortices, alongside curvature abnormalities, including decreases in occipital, temporal, and orbitofrontal regions, and increases in somatomotor, prefrontal, and posterior parietal areas. These structural vulnerabilities are spatially correlated with gene expression profiles enriched for glucose metabolism, mitochondrial function, and post-translational histone modifications [76]. Another study revealed region-specific cortical thickness changes, with caudal regions such as the lateral occipital cortex exhibiting significant atrophy in late-stage PD with an average of 9.1 years of disease duration [77]. Genes associated with the cortical atrophy are enriched in pathways related to mitochondrial translation, mitotic cell cycle regulation, deoxyribonucleic acid damage response, and endoplasmic reticulum–Golgi trafficking [77].

A longitudinal MRI study revealed that the cortical atrophy in PD predominantly progresses in the posterior parietal, temporal, and superior frontal regions over a 4-year follow-up period. Genes most strongly linked to higher atrophy rates are enriched for mitochondrial and metabolic functions [78]. In addition, the posterior cingulate structural covariance network and the anterior cingulate structural covariance network have been associated with gray matter atrophy that exceeded the level of age-related decline in late-stage PD [79]. Functional enrichment showed that genes related to the two networks are involved in neurotransmitter signaling pathways, including G protein‐coupled receptor, GABAergic, glutamatergic, and serotonergic systems, as well as transcriptional regulation and synaptic plasticity. Notably, both networks exhibit high expression of cholinergic marker genes, particularly *NPPA, SOSTDC1*, and *TYRP1* [79]*.*

In conclusion, early-stage structural changes in PD predominantly affect the occipital, temporal, and basal ganglia regions, with progression toward the cingulate, somatomotor, parietal, and frontal cortices over time. These spatial patterns of gray matter vulnerability are linked to distinct molecular mechanisms—early changes are associated with synaptic signaling, while later-stage degeneration involves mitochondrial dysfunction and metabolic stress.

#### Transcriptomic signatures related to non-motor symptoms in PD

The cortical thickness-related gene expression patterns that are also positively associated with Mini-Mental State Examination in PD, are enriched in pathways involving mitochondrial biogenesis, macroautophagy, and endoplasmic reticulum–Golgi transport [77]. PD patients with visual dysfunction, an early marker of dementia risk, show a selective pattern of white matter connectivity loss, which is significantly associated with lower cognitive performance and predominantly affects interhemispheric and subcortical-cortical connections. The interhemispheric loss is linked to the downregulation of genes in the smoothened signaling pathway, enriched in glutamatergic neurons, while the subcortical-cortical loss involves reduced expression of myelination-related genes, enriched in oligodendrocytes. Expression-weighted cell-type enrichment analysis further confirmed distinct cellular contributions: glutamatergic neurons for interhemispheric loss and oligodendrocytes for subcortical-cortical loss, with upregulated genes also enriched in GABAergic neurons [80].

A meta-analysis revealed that PD patients with psychosis exhibit significant grey matter volume reductions in parieto-temporo-occipital regions, including the precuneus, inferior parietal gyrus, inferior occipital gyrus, and middle temporal gyrus, compared to PD patients without psychosis. Spatial transcriptomic analyses showed that the grey matter loss correlated positively with 5-HT2a expression and negatively with 5-HT1a expression, suggesting a differential role of serotonergic signaling in the neuroanatomical vulnerability underlying PD psychosis [81]. In PD patients who experience visual hallucinations, a distinct structural subnetwork of brain regions termed the visual-hippocampal-subnetwork, shows significantly reduced structural connectivity and diminished average controllability, indicating impaired capacity to influence global brain dynamics. This subnetwork includes regions such as the thalamus and temporal cortex. Weighted gene co-expression network analysis identified two gene modules that were significantly associated with the visual-hippocampal-subnetwork: the downregulated module, enriched for genes involved in messenger ribonucleic acid processing and chromatin organization, predominantly expressed in oligodendrocytes; and the upregulated module, enriched for genes related to protein localization and membrane trafficking, primarily expressed in glutamatergic and GABAergic neurons [82].

Imaging transcriptomic studies in PD have elucidated symptom-specific structural and molecular signatures across cognitive decline, psychosis, visual dysfunction, and visual hallucinations. These findings highlight distinct cell-type and pathway-level mechanisms, such as serotonergic imbalance, oligodendrocyte-mediated myelination loss, and glutamatergic/GABAergic dysregulation, offering promising targets for precision therapies for neuropsychiatric and cognitive symptoms in PD. Future imaging transcriptomic studies should incorporate multiple structural metrics and multi-cohort datasets to investigate the underlying mechanisms of specific phenotypes, thereby enhancing reproducibility and generalizability. Symptom coverage remains relatively narrow; expanding research to include both motor and non-motor symptoms, such as freezing of gait, fatigue, and apathy, is essential. Moreover, phenotype dynamics such as the transformation from pre-PD to diagnosed PD, symptom progression and prognosis, and clinical trajectories, warrant further exploration to uncover temporally sensitive molecular drivers of structural vulnerability in PD.

### Imaging transcriptomic analyses using functional modalities in PD

Connectome gradient analysis captures the hierarchical organization of brain networks, reflecting transitions from unimodal sensory regions to transmodal integrative hubs. In PD, functional gradient analysis revealed significantly increased dispersion in networks such as the visual, somatomotor, dorsal attention, frontoparietal, default mode, and subcortical systems compared to healthy controls, indicating disrupted cortical hierarchy [83, 84]. Transcriptome–neuroimaging association analyses revealed that these gradient alterations are spatially linked to gene expression patterns enriched in biological processes such as metal ion and inorganic cation transmembrane transport. The associated genes are overexpressed in Ntsr^+^ and Glt25d2 neurons [83]. Notably, patients with postural instability- and gait disorder-dominant subtype demonstrate unique reductions in the default mode network and limbic network gradient values, along with increased integration in ventral attention and somatomotor networks. These are associated with gene sets enriched in immune-related cell types and Cort^+^ neurons, suggesting a broader molecular dysfunction in this PD subtype [83]. Consistently, FDG-PET imaging showed elevated glucose uptake in the somatomotor and ventral attention networks, but reduced uptake in the visual network in PD. Principal component analysis integrating these two modalities defined a special cortical functional–metabolic architecture of PD, which is spatially associated with expression patterns of genes enriched for metabolic, catabolic, cellular response to ions, and regulation of DNA transcription and RNA biosynthesis [84].

The striatum is a primary region of pathological change in PD. Recent research has identified three distinct striatal functional connectivity gradients in PD. Gradient 1 aligns with anatomical subdivisions, remains stable across disease stages, and shows no significant gene enrichment. Gradient 2 reflects dopaminergic innervation and demonstrates strong spatial correspondence with dopamine transporter availability, suggesting involvement of genes related to dopaminergic signaling, although specific enrichment pathways were not detailed. Gradient 3 is associated with the cortico-striatal connectivity and cognitive impairment, and reveals robust transcriptomic associations. Genes with positive weights are enriched in biological processes such as intracellular protein transport, membrane organization, and proteolysis, with KEGG pathways including mitophagy and PD pathway. Negatively weighted genes are linked to brain development, membrane potential regulation, and chromatin remodeling, with enrichment in cAMP and Rap1 signaling pathways. Therefore, Gradient 3 is a key functional change and molecular interface underlying cognitive decline in PD [85]. In addition, brain regions with higher intrinsic expression of the *MAPT* gene are disproportionately vulnerable to functional connectivity loss in PD. These highly connected “hub” regions, essential for efficient brain network communication, exhibit significant reductions in connection strength, correlated with impaired executive function, particularly verbal fluency. Importantly, the degree of connectivity loss in PD patients is positively associated with *MAPT* expression level, suggesting that the tau-related molecular architecture may predispose specific brain regions to neurodegeneration. In contrast, no significant associations were found with *SNCA* expression, underscoring a distinct network-level impact of tau pathology in PD [86].

### Imaging transcriptomic analyses using specialized modalities in PD

The regional susceptibility to brain lesions in PD may stem from oxidative stress secondary to brain iron deposition. Based on this hypothesis, a previous study has found that genes significantly associated with the spatial pattern of cortical iron deposition in PD are enriched in biological processes related to heavy metal detoxification, synaptic function, and nervous system development, and are primarily expressed in astrocytes and glutamatergic neurons [87].

In a longitudinal multimodal imaging study of tremor-dominant PD patients undergoing magnetic resonance-guided focused ultrasound (MRgFUS) thalamotomy, researchers identified a U-shaped MRgFUS-sensitive subnetwork that was reflective of both the trajectory of hand tremor recovery and the structural remodeling induced by the lesion. Multimodal imaging revealed that changes within this network were accompanied by alterations in cerebral blood flow and gray matter volume. Gene enrichment analyses revealed a strong dopaminergic signature of the affected regions, with elevated expression of genes related to D1/D2 receptors and dopamine transporters. PET imaging confirmed that the subnetwork changes were tightly linked to dopaminergic activity. Together, these findings highlight a dynamic loop between network remodeling and dopamine-related gene expression, offering mechanistic insight into the long-term efficacy of MRgFUS thalamotomy [88].

### Shared and distinct imaging-transcriptomic signatures in AD and PD

Synaptic function is consistently implicated in both disorders. In AD, synaptic signaling genes are significantly related to regions vulnerable to Aβ [23] and tau deposition [31], connectivity alterations [53], and MS changes [42], while in PD, synaptic signaling is repeatedly linked to Braak stage [66] and MS changes [75]. These cross-modalities’ spatial correlations of synaptic function suggest that synapses may represent a shared vulnerability mechanism across diseases.

The immune system and metabolism are also involved in both AD and PD imaging transcriptomic studies. For example, in AD imaging transcriptomic studies, the FDG‑PET hypometabolic regions are enriched for genes linked to mitochondrial dysfunction such as *NDUFS4* [17] and type 2 diabetes risk genes [34], highlighting impaired mitochondrial stability and glucose metabolism. On the other hand, the Aβ‑driven activity changes show enrichment in inflammation and immune system pathways [23]. In PD, immune‑related genes such as *GBA* and *TMEM175* are associated with α‑synuclein propagation [68], and imaging analyses emphasized enrichment in mitophagy [85], mitochondrial and metabolic functions [78].

More importantly, as the abovementioned biological processes are broadly involved in brain biology, future research is needed to refine these findings to more specific sub-processes or even individual genes to disentangle shared neurodegenerative mechanisms from disease-specific molecular drivers. Distinct disease‑specific features are also exhibited. In PD, dopaminergic activity plays a characteristic role, reflecting the selective vulnerability of dopaminergic neurons and their link to motor dysfunction and disease progression.

### Limitations of current studies and minimum reporting checklist

#### Interpretive constraints in imaging transcriptomics

Despite the advantages of brain-wide transcriptomic data, several limitations remain in their current applications. First, using transcriptomic data from neurologically healthy individuals to investigate the molecular effects of intervention may introduce bias in interpretation [57]. One donor brain in the AHBA exhibited gene expression patterns distinct from other brains and showed signs of a prodromal AD state. Use of the data may potentially skew the results. Expression data from healthy brains may dilute the AD-specific transcriptomic alterations in prodromal cases, while the inclusion of such atypical brains may confound efforts to generalize findings to other disease contexts [33]. In addition, the human brain atlas is inherently biased by the variability of gene expression in the human brain. The variability increases with age, which adds further bias to the analysis of age-related neurodegenerative diseases [89]. Given that three donors in the AHBA were aged 49, 55, and 57 years, while the other three were 24, 31, and 39 years, future studies focused on neurodegenerative diseases should consider conducting sensitivity analyses restricted to the three donors aged 49 and above to further establish the robustness of the results.

Second, imaging transcriptomics primarily reveals spatial correlations, and these associations still require verification from other data sources (such as snRNA-seq/scRNA-seq cell-type signatures, spatial transcriptomics, proteomics, and receptor density maps) to confirm AHBA findings. PET shows good consistency with brain-wide transcriptomic maps for markers related to neurons, synapses, and metabolism. Markers for astrocytes and myelin also match expected gene patterns. However, markers linked to the neuroimmune system, like translocator protein and cyclooxygenase-1, show weaker or inconsistent alignment with microglial and astrocytic gene expression in the healthy brain [90]. Previous studies in imaging transcriptomic analyses of AD and PD have revealed significant involvement of immune [28, 41, 66, 83] and inflammatory pathways [28, 61]. However, the specific biological processes require further experimental validation. In addition, results of imaging transcriptomic analyses should be carefully interpreted when posttranscriptional regulation plays a significant role.

#### Methodological constraints of the current application

Spatial autocorrelation represents a central methodological challenge in imaging transcriptomics [91]. Both regional gene expression patterns and neuroimaging measures exhibit strong spatial smoothness and large‑scale gradients across the cortex. As a result, adjacent brain regions tend to show similar values, violating the assumption of independence that underlies conventional correlation‑based analyses. When regional gene expression is directly correlated with imaging phenotypes, these shared spatial gradients can inflate statistical associations. Without appropriate correction, spatial autocorrelation would compromise the interpretability and reproducibility of imaging–transcriptomic findings [91].

To address this issue, recent methodological advances have introduced replicable approaches such as a spatially informed null model that preserves the intrinsic spatial structure of brain maps while providing more rigorous significance testing [92]. In imaging transcriptomics, spatially constrained null models can be broadly divided into two main types. Non‑parametric spatial permutation models are particularly well-suited for cortical analyses, as the cortical surface can be mapped to a sphere and rotated to shuffle values while preserving spatial autocorrelation. Parameterized spatial models are more appropriate for subcortical and voxel‑level data, where spherical projection is not feasible; these models estimate the intrinsic spatial autocorrelation of empirical maps and generate surrogate maps with randomized topography but similar spatial structure. There is no perfect null model; each approach has limitations, and error rates can remain high for strongly autocorrelated data. The choice of framework should therefore be tailored to the research context, including parcellation scheme, resolution, and anatomical domain. For detailed comparisons and implementation guidelines, see prior work [91].

While PLS regression has become a cornerstone in imaging transcriptomics for linking spatially resolved gene expression from the AHBA with neuroimage phenotypes, its interpretability is fraught with subtle yet critical pitfalls. First, gene loadings may be misinterpreted as direct measures of biological importance; however, in high-dimensional transcriptomic data, multicollinearity can disperse weights across correlated genes or inflate noise-driven features. Such challenges are best addressed through rigorous statistical validation. Second, the stability of selected gene signatures is frequently overlooked; without rigorous bootstrap resampling, identified markers may reflect sample-specific noise rather than reproducible biological signals, particularly given the limited sample sizes typical of AHBA studies. Third, improper cross-validation strategies—such as failing to account for spatial autocorrelation or leaking preprocessing steps across training and test folds—can lead to severely optimistic performance estimates and spurious associations. Finally, component selection based solely on explained variance risks overfitting noise; instead, components should be validated via permutation testing and predictive ability to ensure they capture genuine covariance structures [59]. Critically, none of these limitations are inherent flaws of PLS itself, but rather consequences of its misapplication to high-dimensional, spatially structured data. Each can be effectively mitigated through methodologically sound practices, including bootstrap resampling to assess stability, spatially aware cross-validation to prevent data leakage, and permutation testing to establish statistical significance. Therefore, PLS remains a powerful and reliable tool for hypothesis generation.

#### Minimum reporting checklist for applied AHBA in neurological diseases

Previous studies have provided some toolboxes of the three key steps of Imaging Transcriptomic analyses [5]. To promote quality assessment and heterogeneity evaluation in imaging transcriptomic research, and to facilitate reproducibility and cross‑study hypothesis validation, we propose a Minimum Reporting Checklist (Table 1) based on previous methodological work. The checklist is organized into three main components: (1) AHBA data preprocessing and relating expression with other modality brain data, (2) identifying the spatial correlation between genes and neuroimages (if applicable), and (3) gene enrichment analyses (if applicable). Most importantly, we recommend public availability of codes in future studies to enable cross‑study validation.

**Table 1:** Minimum reporting checklist for applied AHBA in neurological diseases

Item	Recommendation and report
*Part A. AHBA data preprocessing and relating expression with other modality brain data*	It is recommended to use established preprocessing toolboxes, such as abagen
1 Annotation	Annotation for genes should be clearly described, including the choice of database and the time of annotation
2 Transforming probe-level data into gene-level data	Mapping process from raw probes to genes should be explicitly reported. At a minimum, this should include the probe-level denoising or filtering strategy, and the method used to match probes to genes
2.1 Probe filtering	
2.2 Probe selection	
2.3 Expression value	
3 Sample assignment	The approach to mapping gene expression levels to brain modalities should be explained. This should cover the parcellation scheme, the selection of mapped regions (e.g., left vs. right hemisphere, subcortical vs. whole brain), the distance threshold applied, and the strategy for handling inter-donor variability and whether any donor is excluded
3.1 Parcellations	
3.2 Hemisphere handling	
3.3 Brain structures	
3.4 Distance threshold	
3.5 Data normalization	
3.6 Gene filtering	Authors should state whether further filtering of genes was performed after initial inclusion
*Part B. Identifying the spatial correlation between genes and neuroimages*	For studies conducting spatial correlation analyses and statistical significance testing, it is essential to detail processes of spatial correlation model construction and statistical significance assessment, and whether spatial autocorrelation is considered in this step
4.1 Detailed correlating methods	
4.2 Testing of significance	
4.3 Accounting for spatial effects in testing of significance	
*Part C. Gene enrichment analyses*	For studies employing gene enrichment analyses to support biological interpretation, enrichment method should be described, along with whether gene–gene coexpression and spatial effect are accounted for in this step
5.1 Detailed methods of enrichment analyses and test of significance	
5.2 Accounting for gene–gene coexpression and spatial effect in gene enrichment methods	
*Part D. Others*	
6 Characteristics of included human participants	If new human participants are included, authors should report the details of the demographic features of the participants. For patient participants, the disease-related clinical characteristics should also be reported in detail
7 Imaging modalities	Studies involving neuroimaging data should follow established neuroimaging reporting guidelines and provide a detailed description of imaging data processing. Ideally, raw imaging data and code should be made available
8 Aim of the study	As the application of the AHBA data can be diverse, authors should detail the aims of studies
9 Code availability	To ensure reproducibility, the code used for data processing and analysis should be shared whenever possible
9.1 Code deposit URL	

AHBA, Allen human brain atlas

## Future perspectives

The imaging transcriptomics methodologies hold great promise for exploring the molecular underpinnings of a wide range of clinical questions, such as the biological mechanisms associated with different stages of AD under the ATN framework [93], transcriptomic signatures linked to varying progression rates in AD and PD, and the molecular basis of non-motor symptoms such as depression, anxiety, and apathy in these patients. Such integrative data-driven approaches will deepen our understanding, ultimately contributing to the resolution of clinically relevant challenges [94].

Integrating brain-wide transcriptomic atlases with multimodal and multilayer neurobiological datasets offers a more comprehensive framework for exploring region-specific vulnerability and pathological mechanisms in neurodegenerative diseases. Brain-wide transcriptomic analyses support the previously identified pan-neurodegenerative mechanism of regional vulnerability revealed by transcriptomic analyses of localized brain tissues from patients [95]. These studies expanded the previous result of differential transcriptomic profiles of Braak stages based on PD frontal cortex examples [96]. Integrative analyses of AHBA and regionally sampled transcriptomic datasets of patients (e.g., Mount Sinai, Mayo Clinic, ROSMAP) [88, 97] enable cross-modal analysis and further confirmation of spatial gene expression patterns in corresponding patients [98]. Moreover, as brain-wide transcriptomic data provide information at the transcriptional level, in the future, integration of multiple resources such as brain protein atlases and methylation maps can be made to explore both transcriptional and post-transcriptional mechanisms underlying brain changes in neurodegenerative disorders [99, 100].

In addition, although current single‑cell and spatial transcriptomic technologies provide substantially higher spatial resolution than neuroimaging, it is difficult to align the single‑cell transcriptomic atlas with neuroimages at comparable spatial scales in humans. Current studies are beginning to overcome this limitation [101]. The integration of single‑cell and spatial transcriptomic maps with standardized neuroimaging coordinate frameworks has already been established in the mouse brain using the Allen CCF, and similar cross‑scale alignment is expected to become feasible in humans [102]. Emerging whole‑brain spatial and single‑cell transcriptomic atlases—built using spatial transcriptomics, single‑cell and single‑nucleus RNA sequencing, and other cell‑level multi‑omic approaches, can be registered to the Montreal Neurological Institute (MNI) space in the future. These next‑generation atlases will enable true multiscale integration, allowing cellular and molecular signatures to be mapped directly onto macroscale imaging phenotypes. Continued development of atlases with markedly higher spatial and temporal resolution will further expand the utility of brain‑wide transcriptomic resources, supporting more precise identification of drug targets, disease‑associated genes, and other mechanistic pathways.

Future applications will also require more rigorous validation frameworks to ensure that imaging transcriptomic findings are robust, reproducible, and biologically meaningful. Cross‑atlas replication will be essential, including testing whether spatial gene–imaging associations derived from one transcriptomic atlas (e.g., AHBA) can be reproduced in independent transcriptomic atlases. Validation across independent neuroimaging cohorts will further strengthen confidence in neuroimaging phenotype-based correlation analysis, particularly for disease‑related spatial patterns that may vary with sample characteristics, disease stage, or imaging modality. Another important direction is assessing the robustness of results to different brain parcellation schemes and spatial resolutions, given that regional definitions can substantially influence spatial correlation analyses. We also anticipate that future studies will further refine the identified gene sets through more stringent filtering and direct biological experimentation, enabling stronger mechanistic validation of imaging transcriptomic discoveries. Together, these validation strategies will help establish a more reliable foundation for translating imaging transcriptomics into mechanistic insights and clinically actionable knowledge.

## Conclusion

AHBA provides high-resolution brain-wide microarray data, enabling the spatially precise mapping of gene expression across anatomically defined regions. The comprehensive probe coverage and standardized data format of AHBA support robust statistical comparisons and protein interaction modeling, making it a powerful resource for uncovering disease-relevant molecular networks in the human brain. This review systematically summarizes the application of brain-wide transcriptomic data—particularly the AHBA—in exploring regional susceptibility, pathological progression patterns, and molecular mechanisms of imaging phenotypes in AD and PD. Pathways related to synaptic signaling, neurotransmission, metabolism, and immunity play central roles in AD and PD. Evidence shows that specific gene expression patterns are closely associated with the spatial distribution of neuroimaging features, providing a molecular basis for understanding region-specific vulnerability. With the continuous optimization of multimodal data integration methods and the ongoing expansion of methods utilizing brain-wide transcriptomic resources, brain-wide transcriptomic data hold great promise for uncovering the region-specific molecular mechanisms of neurodegenerative diseases.

## Supplementary Information

Additional file 1. Methods.

Additional file 2. **Table S1**. Detailed information of the 60 studies included in this review.

Additional file 3. **Table S2**. Critical appraisal of papers included in the systematic review.

## Data Availability

No datasets were generated or analysed during the current study.

## Abbreviations and glossary

AHBA (Allen Human Brain Atlas): A comprehensive, high-resolution map of gene expression across the entire adult human brain, based on microarray data from postmortem donors.
AD (Alzheimer’s Disease): The most common neurodegenerative disorder, characterized by progressive memory loss, cognitive decline, and the accumulation of amyloid-beta plaques and tau tangles in the brain.
PD (Parkinson’s Disease): A chronic neurodegenerative disorder primarily affecting movement, caused by the loss of dopaminergic neurons and the presence of alpha-synuclein aggregates (Lewy bodies).
PLS (Partial Least Squares): A statistical method used to find relationships between two datasets (e.g., gene expression and brain imaging) by identifying latent variables that maximize their covariance. Commonly used in imaging transcriptomics.
FDG-PET (Fluorodeoxyglucose Positron Emission Tomography): An imaging technique that measures glucose metabolism in the brain. Reduced FDG uptake often indicates neuronal dysfunction or degeneration in neurodegenerative diseases.
MS (Morphometric Similarity): A functional imaging technique that uses radioactive tracers to visualize metabolic processes, protein deposition (e.g., amyloid, tau), or neurotransmitter receptor density in the living brain.
MSN (Morphometric Similarity Network): A whole-brain network constructed by calculating morphometric similarity between all pairs of brain regions, revealing hierarchical organization.
R2SN (Regional Radiomics Similarity Network): A novel macroscale structural measure based on radiomic features extracted from MRI, capturing hierarchical cortical organization.
DMN (Default Mode Network): A large-scale brain network active during rest and self-referential thought, including regions like the posterior cingulate cortex and medial prefrontal cortex.
rTMS (Repetitive Transcranial Magnetic Stimulation): A non-invasive brain stimulation technique that uses magnetic fields to modulate neural activity in targeted cortical regions, explored as a potential therapy for neurological and psychiatric disorders.
PET (Positron Emission Tomography): A functional imaging technique that uses radioactive tracers to visualize metabolic processes, protein deposition (e.g., amyloid, tau), or neurotransmitter receptor density in the living brain.
MRI (Magnetic Resonance Imaging): A non-invasive medical imaging technique that uses strong magnetic fields and radio waves to generate detailed images of the body's internal structures.

## References

[1] Mroczek M, Desouky A, Sirry W. Imaging transcriptomics in neurodegenerative diseases. J Neuroimaging. 2021;31:244–50. 33368775 10.1111/jon.12827

[2] Hawrylycz MJ, Lein ES, Guillozet-Bongaarts AL, Shen EH, Ng L, Miller JA, et al. An anatomically comprehensive atlas of the adult human brain transcriptome. Nature. 2012;489:391–9. 22996553 10.1038/nature11405 PMC4243026

[3] Kang HJ, Kawasawa YI, Cheng F, Zhu Y, Xu X, Li M, et al. Spatio-temporal transcriptome of the human brain. Nature. 2011;478:483–9. 22031440 10.1038/nature10523 PMC3566780

[4] Selvaggi P, Rizzo G, Mehta MA, Turkheimer FE, Veronese M. Integration of human whole-brain transcriptome and neuroimaging data: practical considerations of current available methods. J Neurosci Methods. 2021;355:109128. 33722642 10.1016/j.jneumeth.2021.109128

[5] Arnatkeviciute A, Markello RD, Fulcher BD, Misic B, Fornito A. Toward best practices for imaging transcriptomics of the human brain. Biol Psychiatry. 2023;93:391–404. 36725139 10.1016/j.biopsych.2022.10.016

[6] Arnatkeviciute A, Fulcher BD, Bellgrove MA, Fornito A. Imaging transcriptomics of brain disorders. Biol Psychiatry Glob Open Sci. 2022;2:319–31. 36324650 10.1016/j.bpsgos.2021.10.002 PMC9616271

[7] Arnatkevic̆iūtė A, Fulcher BD, Fornito A. A practical guide to linking brain-wide gene expression and neuroimaging data. Neuroimage. 2019;189:353–67. 30648605 10.1016/j.neuroimage.2019.01.011

[8] Fornito A, Arnatkevičiūtė A, Fulcher BD. Bridging the gap between connectome and transcriptome. Trends Cogn Sci. 2019;23:34–50. 30455082 10.1016/j.tics.2018.10.005

[9] Giacomel A, Martins D, Frigo M, Turkheimer F, Williams SCR, Dipasquale O, et al. Integrating neuroimaging and gene expression data using the imaging transcriptomics toolbox. STAR Protocols. 2022;3:101315. 35479111 10.1016/j.xpro.2022.101315 PMC9036395

[10] Markello RD, Arnatkeviciute A, Poline J-B, Fulcher BD, Fornito A, Misic B. Standardizing workflows in imaging transcriptomics with the abagen toolbox. Elife. 2021;10:e72129. 34783653 10.7554/eLife.72129 PMC8660024

[11] Liu Z, Rolls ET, Liu Z, Zhang K, Yang M, Du J, et al. Brain annotation toolbox: exploring the functional and genetic associations of neuroimaging results. Bioinformatics. 2019;35:3771–8. 30854545 10.1093/bioinformatics/btz128

[12] Pak V, Adewale Q, Bzdok D, Dadar M, Zeighami Y, Iturria-Medina Y. Distinctive whole-brain cell types predict tissue damage patterns in thirteen neurodegenerative conditions. Elife. 2024;12:RP89368. 38512130 10.7554/eLife.89368 PMC10957173

[13] Huang Y, Li Y, Pan H, Han L. Global, regional, and national burden of neurological disorders in 204 countries and territories worldwide. J Glob Health. 2023;13:04160. 38018250 10.7189/jogh.13.04160 PMC10685084

[14] Ganguly G, Chakrabarti S, Chatterjee U, Saso L. Proteinopathy, oxidative stress and mitochondrial dysfunction: cross talk in Alzheimer’s disease and Parkinson’s disease. DDDT. 2017;11:797–810. 28352155 10.2147/DDDT.S130514 PMC5358994

[15] Compta Y, Revesz T. Neuropathological and biomarker findings in Parkinson’s disease and Alzheimer’s disease: from protein aggregates to synaptic dysfunction. J Parkinsons Dis. 2021;11:107–21. 33325398 10.3233/JPD-202323 PMC7990431

[16] Yu M, Risacher SL, Nho KT, Wen Q, Oblak AL, Unverzagt FW, et al. Spatial transcriptomic patterns underlying amyloid-β and tau pathology are associated with cognitive dysfunction in Alzheimer’s disease. Cell Rep. 2024;43:113691. 38244198 10.1016/j.celrep.2024.113691 PMC10926093

[17] Mullins R, Kapogiannis D. Alzheimer’s disease-related genes identified by linking spatial patterns of pathology and gene expression. Front Neurosci. 2022;16:908650. 35774552 10.3389/fnins.2022.908650 PMC9237461

[18] Yao J, Li Z, Zhou Z, Bao A, Wang Z, Wei H, et al. Distinct regional vulnerability to aβ and iron accumulation in *post mortem* AD brains. Alzheimer Dement. 2024;20:6984–97. 10.1002/alz.14188 PMC11485316 39175425

[19] Mattsson N, Palmqvist S, Stomrud E, Vogel J, Hansson O. Staging β-amyloid pathology with amyloid positron emission tomography. JAMA Neurol. 2019;76:1319. 31314895 10.1001/jamaneurol.2019.2214 PMC6646987

[20] Sepulcre J, Grothe MJ, d’Oleire Uquillas F, Ortiz-Terán L, Diez I, Yang H-S, et al. Neurogenetic contributions to amyloid beta and tau spreading in the human cortex. Nat Med. 2018;24:1910–8. 30374196 10.1038/s41591-018-0206-4 PMC6518398

[21] Wojtas AM, Kang SS, Olley BM, Gatherer M, Shinohara M, Lozano PA, et al. Loss of clusterin shifts amyloid deposition to the cerebrovasculature via disruption of perivascular drainage pathways. Proc Natl Acad Sci U S A. 2017;114(33):E6962–71. 28701379 10.1073/pnas.1701137114 PMC5565413

[22] Chang M, Krüssel S, Parajuli LK, Kim J, Lee D, Merodio A, et al. Intercellular communication in the brain through a dendritic nanotubular network. Science. 2025;390:eadr7403. 41037599 10.1126/science.adr7403

[23] Sanchez-Rodriguez LM, Khan AF, Adewale Q, Bezgin G, Therriault J, Fernandez-Arias J, et al. In-vivo neuronal dysfunction by Aβ and tau overlaps with brain-wide inflammatory mechanisms in Alzheimer’s disease. Front Aging Neurosci. 2024;16:1383163. 38966801 10.3389/fnagi.2024.1383163 PMC11223503

[24] Baik JY, Kim M, Bao J, Long Q, Shen L. Alzheimer’s disease neuroimaging initiative identifying Alzheimer’s genes via brain transcriptome mapping. BMC Med Genomics. 2022;15:116. 35590321 10.1186/s12920-022-01260-6 PMC9118564

[25] Florentinus-Mefailoski A, Bowden P, Scheltens P, Killestein J, Teunissen C, Marshall JG. The plasma peptides of Alzheimer’s disease. Clin Proteom. 2021;18:17. 10.1186/s12014-021-09320-2 PMC8240224 34182925

[26] Moreno-Madrid I, Romero-Imbroda J, Reyes-Bueno JA, Arrabal-Gómez C, Díaz-Casares A, Díaz-Sanchez E, et al. Peripheral expression of NPY1R-based heteroreceptor complexes reflects hippocampal neuroimmune status: novel biomarkers for early alzheimer’s disease detection. Biomed Pharmacother. 2025;189:118332. 40618588 10.1016/j.biopha.2025.118332

[27] Nahalka J. The role of the protein–RNA recognition code in neurodegeneration. Cell Mol Life Sci. 2019;76:2043–58. 30980111 10.1007/s00018-019-03096-3 PMC11105320

[28] Anand C, Abdelnour F, Sipes B, Ma D, Maia PD, Torok J, et al. Selective vulnerability and resilience to Alzheimer’s disease tauopathy as a function of genes and the connectome. Brain. 2025;148:3679–93. 40631882 10.1093/brain/awaf179 PMC12493037

[29] Zheng L, Rubinski A, Denecke J, Luan Y, Smith R, Strandberg O, et al. Combined connectomics, MAPT gene expression, and amyloid deposition to explain regional tau deposition in alzheimer disease. Ann Neurol. 2024;95:274–87. 37837382 10.1002/ana.26818

[30] Montal V, Diez I, Kim C-M, Orwig W, Bueichekú E, Gutiérrez-Zúñiga R, et al. Network tau spreading is vulnerable to the expression gradients of *APOE* and glutamatergic-related genes. Science Translational Medicine. 2022;14:eabn7273. 35895837 10.1126/scitranslmed.abn7273 PMC9942690

[31] Luan Y, Zheng L, Denecke J, Dehsarvi A, Roemer-Cassiano SN, Dewenter A, et al. Multimodal spatial gradients to explain regional susceptibility to fibrillar tau in Alzheimer’s disease. Alzheimer Dement. 2025;21:e70170. 10.1002/alz.70170 PMC12060132 40342276

[32] Ardanaz CG, Ramírez MJ, Solas M. Brain metabolic alterations in Alzheimer’s disease. IJMS. 2022;23:3785. 35409145 10.3390/ijms23073785 PMC8998942

[33] Patel S, Howard D, Man A, Schwartz D, Jee J, Felsky D, et al. Donor-specific transcriptomic analysis of Alzheimer’s disease-associated hypometabolism highlights a unique donor, ribosomal proteins and microglia. eneuro. 2020;7:0255–320. 10.1523/ENEURO.0255-20.2020 PMC7772516 33234543

[34] Nugent S, Potvin O, Cunnane SC, Chen T-H, Duchesne S. Associating type 2 diabetes risk factor genes and FDG-PET brain metabolism in normal aging and Alzheimer’s disease. Front Aging Neurosci. 2020;12:580633. 33192474 10.3389/fnagi.2020.580633 PMC7661639

[35] Mullins RJ, Mustapic M, Goetzl EJ, Kapogiannis D. Exosomal biomarkers of brain insulin resistance associated with regional atrophy in Alzheimer’s disease. Human Brain Mapp. 2017;38:1933–40. 10.1002/hbm.23494 PMC5342917 28105773

[36] Mullins RJ, Diehl TC, Chia CW, Kapogiannis D. Insulin resistance as a link between amyloid-beta and tau pathologies in Alzheimer’s disease. Front Aging Neurosci. 2017;9:118. 28515688 10.3389/fnagi.2017.00118 PMC5413582

[37] Tang X, Guo Z, Chen G, Sun S, Xiao S, Chen P, et al. A multimodal meta-analytical evidence of functional and structural brain abnormalities across Alzheimer’s disease spectrum. Ageing Res Rev. 2024;95:102240. 38395200 10.1016/j.arr.2024.102240

[38] Groot C, Grothe MJ, Mukherjee S, Jelistratova I, Jansen I, van Loenhoud AC, et al. Differential patterns of gray matter volumes and associated gene expression profiles in cognitively-defined Alzheimer’s disease subgroups. NeuroImage Clin. 2021;30:102660. 33895633 10.1016/j.nicl.2021.102660 PMC8186562

[39] Li Y, Zhou G, Peng J, Liu L, Zhang F, Iturria-Medina Y, et al. White matter dysfunction in Alzheimer’s disease is associated with disease-related transcriptomic signatures. Commun Biol. 2025;8:820. 40437109 10.1038/s42003-025-08177-7 PMC12120127

[40] Yu H, Ding Y, Wei Y, Dyrba M, Wang D, Kang X, et al. Morphological connectivity differences in Alzheimer’s disease correlate with gene transcription and cell-type. Hum Brain Mapp. 2023;44:6364–74. 37846762 10.1002/hbm.26512 PMC10681645

[41] Brusini L, Dolci G, Pini L, Cruciani F, Pizzagalli F, Provero P, et al. Morphometric similarity patterning of amyloid-β and tau proteins correlates with transcriptomics in the Alzheimer’s disease continuum. IJMS. 2024;25:12871. 39684582 10.3390/ijms252312871 PMC11641379

[42] Zhang Y, Ma M, Xie Z, Wu H, Zhang N, Shen J. Bridging the gap between morphometric similarity mapping and gene transcription in Alzheimer’s disease. Front Neurosci. 2021;15:731292. 34671240 10.3389/fnins.2021.731292 PMC8522649

[43] Peng J, Tang Q, Li Y, Liu L, Biswal BB, Wang P. Neuromorphic deviations associated with transcriptomic expression and specific cell type in Alzheimer’s disease. Sci Rep. 2025;15:7460. 40032887 10.1038/s41598-025-90872-w PMC11876660

[44] Zheng C, Zhao W, Yang Z, Guo S. Dysfunction in the hierarchy of morphometric similarity network in Alzheimer’s disease and its correlation with cognitive performance and gene expression profiles. Psychol Med. 2025;55:e42. 39934009 10.1017/S0033291725000091 PMC12055026

[45] Zhao K, Wang D, Wang D, Chen P, Wei Y, Tu L, et al. Macroscale connectome topographical structure reveals the biomechanisms of brain dysfunction in Alzheimer’s disease. Sci Adv. 2024;10:eado8837. 39392880 10.1126/sciadv.ado8837 PMC11809497

[46] Park B, Lee W, Han K. Modeling the interactions of alzheimer-related genes from the whole brain microarray data and diffusion tensor images of human brain. BMC Bioinform. 2012;13:S10. 10.1186/1471-2105-13-S7-S10 PMC3348019 22594996

[47] Lu Y, Zhang X, Hu L, Cheng Q, Zhang Z, Zhang H, et al. Consistent genes associated with structural changes in clinical Alzheimer’s disease spectrum. Front Neurosci. 2024;18:1376288. 39554844 10.3389/fnins.2024.1376288 PMC11564164

[48] Zhang Y, Sloan SA, Clarke LE, Caneda C, Plaza CA, Blumenthal PD, et al. Purification and characterization of progenitor and mature human astrocytes reveals transcriptional and functional differences with mouse. Neuron. 2016;89:37–53. 26687838 10.1016/j.neuron.2015.11.013 PMC4707064

[49] Lake BB, Chen S, Sos BC, Fan J, Kaeser GE, Yung YC, et al. Integrative single-cell analysis of transcriptional and epigenetic states in the human adult brain. Nat Biotechnol. 2018;36:70–80. 29227469 10.1038/nbt.4038 PMC5951394

[50] Habib N, Avraham-Davidi I, Basu A, Burks T, Shekhar K, Hofree M, et al. Massively parallel single-nucleus RNA-seq with DroNc-seq. Nat Methods. 2017;14:955–8. 28846088 10.1038/nmeth.4407 PMC5623139

[51] Darmanis S, Sloan SA, Zhang Y, Enge M, Caneda C, Shuer LM, et al. A survey of human brain transcriptome diversity at the single cell level. Proc Natl Acad Sci USA. 2015;112:7285–90. 26060301 10.1073/pnas.1507125112 PMC4466750

[52] Li M, Santpere G, Imamura Kawasawa Y, Evgrafov OV, Gulden FO, Pochareddy S, et al. Integrative functional genomic analysis of human brain development and neuropsychiatric risks. Science. 2018;362:eaat7615. 30545854 10.1126/science.aat7615 PMC6413317

[53] He B, Gorijala P, Xie L, Cao S, Yan J. Gene co-expression changes underlying the functional connectomic alterations in Alzheimer’s disease. BMC Med Genomics. 2022;15:92. 35461274 10.1186/s12920-022-01244-6 PMC9035246

[54] Wang D, Li Z, Zhao K, Chen P, Yang F, Yao H, et al. Macroscale gradient dysfunction in Alzheimer’s disease: patterns with cognition terms and gene expression profiles. Hum Brain Map. 2024;45:e70046. 10.1002/hbm.70046 PMC11502409 39449114

[55] Zheng C, Zhao W, Yang Z, Guo S. Functional connectome hierarchy dysfunction in Alzheimer’s disease and its relationship with cognition and gene expression profiling. J Neurosci Res. 2024;102:e25280. 38284860 10.1002/jnr.25280

[56] Zheng C, Xiao X, Zhao W, Yang Z, Guo S. Functional brain network controllability dysfunction in Alzheimer’s disease and its relationship with cognition and gene expression profiling. J Neural Eng. 2024;21:026018. 10.1088/1741-2552/ad357e 38502960

[57] Yao W, Hou X, Zheng W, Shi X, Zhang J, Bai F. Brain overlapping system-level architecture influenced by external magnetic stimulation and internal gene expression in AD-spectrum patients. Mol Psychiatry. 2025;30:4110–21. 40185902 10.1038/s41380-025-02991-5

[58] Dusek P, Hofer T, Alexander J, Roos PM, Aaseth JO. Cerebral iron deposition in neurodegeneration. Biomolecules. 2022;12:714. 35625641 10.3390/biom12050714 PMC9138489

[59] Yang A, Luan J, Xu M, Du L, Lv K, Hu P, et al. Regional brain iron correlates with transcriptional and cellular signatures in Alzheimer’s disease. Alzheimer Dement. 2025;21:e14459. 10.1002/alz.14459 PMC11775454 39876820

[60] Chu J, Yao J, Li Z, Li J, Zhang Y, Liu C, et al. Brain tissue electrical conductivity as a promising biomarker for dementia assessment using MRI. Alzheimer Dement. 2025;21:e70270. 10.1002/alz.70270 PMC12185248 40551292

[61] Adewale Q, Khan AF, Carbonell F, Iturria-Medina Y. Integrated transcriptomic and neuroimaging brain model decodes biological mechanisms in aging and Alzheimer’s disease. Elife. 2021;10:e62589. 34002691 10.7554/eLife.62589 PMC8131100

[62] Lancour D, Dupuis J, Mayeux R, Haines JL, Pericak-Vance MA, Schellenberg GC, et al. Analysis of brain region-specific co-expression networks reveals clustering of established and novel genes associated with Alzheimer disease. Alzheimer Res Ther. 2020;12:103. 10.1186/s13195-020-00674-7 PMC7469336 32878640

[63] Wang Y-T, Huang C-C, Lin Y-S, Huang W-F, Yang C-Y, Lee C-C, et al. Conditional deletion of eps8 reduces hippocampal synaptic plasticity and impairs cognitive function. Neuropharmacology. 2017;112:113–23. 27450093 10.1016/j.neuropharm.2016.07.021

[64] Petyuk VA, Chang R, Ramirez-Restrepo M, Beckmann ND, Henrion MYR, Piehowski PD, et al. The human brainome: network analysis identifies HSPA2 as a novel Alzheimer’s disease target. Brain. 2018;141:2721–39. 30137212 10.1093/brain/awy215 PMC6136080

[65] Xie L, He B, Varathan P, Nho K, Risacher SL, Saykin AJ, et al. Integrative-omics for discovery of network-level disease biomarkers: a case study in Alzheimer’s disease. Brief Bioinform. 2021;22:bbab21. 10.1093/bib/bbab121 PMC8574309 33971669

[66] Keo A, Mahfouz A, Ingrassia AMT, Meneboo J-P, Villenet C, Mutez E, et al. Transcriptomic signatures of brain regional vulnerability to Parkinson’s disease. Commun Biol. 2020;3:101. 32139796 10.1038/s42003-020-0804-9 PMC7058608

[67] Guo JL, Lee VMY. Cell-to-cell transmission of pathogenic proteins in neurodegenerative diseases. Nat Med. 2014;20:130–8. 24504409 10.1038/nm.3457 PMC4011661

[68] Freeze B, Pandya S, Zeighami Y, Raj A. Regional transcriptional architecture of Parkinson’s disease pathogenesis and network spread. Brain. 2019;142:3072–85. 31359041 10.1093/brain/awz223 PMC8979338

[69] Basaia S, Agosta F, Diez I, Bueichekú E, d’Oleire Uquillas F, Delgado-Alvarado M, et al. Neurogenetic traits outline vulnerability to cortical disruption in Parkinson’s disease. NeuroImage Clinical. 2022;33:102941. 35091253 10.1016/j.nicl.2022.102941 PMC8800137

[70] Zheng Y-Q, Zhang Y, Yau Y, Zeighami Y, Larcher K, Misic B, et al. Local vulnerability and global connectivity jointly shape neurodegenerative disease propagation. PLoS Biol. 2019;17:e3000495. 31751329 10.1371/journal.pbio.3000495 PMC6894889

[71] Yan W, Ye C, Wang T, Sun J, Wu T, Ma T. Misfolded protein propagation in an integrated computational model of structural network and LRRK2 gene expression. Annu Int Conf IEEE Eng Med Biol Soc. 2020;2020:2368–71. 33018482 10.1109/EMBC44109.2020.9178266

[72] Dadgar-Kiani E, Bieri G, Melki R, Gitler AD, Lee JH. Mesoscale connections and gene expression empower whole-brain modeling of α-synuclein spread, aggregation, and decay dynamics. Cell Rep. 2022;41:111631. 36351406 10.1016/j.celrep.2022.111631 PMC10840492

[73] Ji Y, Xu M, Zhao H, Cai H, Chen K, Zhang L, et al. Genetic mechanisms underlying gray matter atrophy in Parkinson’s disease: a combined transcriptome and neuroimaging study. Cereb Cortex. 2025;35:bhaf097. 40302614 10.1093/cercor/bhaf097

[74] Freeze B, Acosta D, Pandya S, Zhao Y, Raj A. Regional expression of genes mediating trans-synaptic alpha-synuclein transfer predicts regional atrophy in Parkinson disease. NeuroImage Clinical. 2018;18:456–66. 29868450 10.1016/j.nicl.2018.01.009 PMC5984599

[75] Wang Y, Xiao Y, Xing Y, Yu M, Wang X, Ren J, et al. Morphometric similarity differences in drug-naive Parkinson’s disease correlate with transcriptomic signatures. CNS Neurosci Ther. 2024;30:e14680. 38529533 10.1111/cns.14680 PMC10964038

[76] Yan S, Lu J, Duan B, Zhu H, Tian T, Qin Y, et al. Genetic and neurochemical profiles underlying cortical morphometric vulnerability to Parkinson’s disease. Brain Res Bull. 2025;221:111222. 39855312 10.1016/j.brainresbull.2025.111222

[77] Keo A, Dzyubachyk O, Van Der Grond J, Van Hilten JJ, Reinders MJT, Mahfouz A. Transcriptomic signatures associated with regional cortical thickness changes in Parkinson’s disease. Front Neurosci. 2021;15:733501. 34658772 10.3389/fnins.2021.733501 PMC8519261

[78] Vo A, Tremblay C, Rahayel S, Shafiei G, Hansen JY, Yau Y, et al. Network connectivity and local transcriptomic vulnerability underpin cortical atrophy progression in Parkinson’s disease. Neuroimage. 2023;40:103523. 38016407 10.1016/j.nicl.2023.103523 PMC10687705

[79] Keo A, Dzyubachyk O, Van Der Grond J, Hafkemeijer A, Van De Berg WDJ, Van Hilten JJ, et al. Cingulate networks associated with gray matter loss in Parkinson’s disease show high expression of cholinergic genes in the healthy brain. Eur J Neurosci. 2021;53:3727–39. 33792979 10.1111/ejn.15216 PMC8251922

[80] Zarkali A, McColgan P, Ryten M, Reynolds RH, Leyland L-A, Lees AJ, et al. Dementia risk in Parkinson’s disease is associated with interhemispheric connectivity loss and determined by regional gene expression. Neuroimage. 2020;28:102470. 33395965 10.1016/j.nicl.2020.102470 PMC7581968

[81] Pisani S, Gunasekera B, Lu Y, Vignando M, Ffytche D, Aarsland D, et al. Grey matter volume loss in Parkinson’s disease psychosis and its relationship with serotonergic gene expression: a meta-analysis. Neurosci Biobehav Rev. 2023;147:105081. 36775084 10.1016/j.neubiorev.2023.105081

[82] Zarkali A, McColgan P, Ryten M, Reynolds R, Leyland L-A, Lees AJ, et al. Differences in network controllability and regional gene expression underlie hallucinations in Parkinson’s disease. Brain. 2020;143:3435–48. 33118028 10.1093/brain/awaa270 PMC7719028

[83] Bu S, Li X, Pang H, Zhao M, Wang J, Liu Y, et al. Motor functional hierarchical organization of cerebrum and its underlying genetic architecture in Parkinson’s disease. J Neurosci. 2025;45:e1492242024. 39824632 10.1523/JNEUROSCI.1492-24.2024 PMC11823334

[84] Zang Z, Zhang X, Song T, Li J, Nie B, Mei S, et al. Association between gene expression and functional-metabolic architecture in Parkinson’s disease. Hum Brain Map. 2023;44:5387–401. 10.1002/hbm.26443 PMC10543112 37605831

[85] Li X, Bu S, Pang H, Yu H, Zhao M, Wang J, et al. Mapping striatal functional gradients and associated gene expression in Parkinson’s disease with continuous cognitive impairment. npj Parkinson’s Dis. 2025;11:138. 40425604 10.1038/s41531-025-01002-2 PMC12117062

[86] Rittman T, Rubinov M, Vértes PE, Patel AX, Ginestet CE, Ghosh BCP, et al. Regional expression of the MAPT gene is associated with loss of hubs in brain networks and cognitive impairment in Parkinson disease and progressive supranuclear palsy. Neurobiol Aging. 2016;48:153–60. 27697694 10.1016/j.neurobiolaging.2016.09.001 PMC5096886

[87] Thomas GEC, Zarkali A, Ryten M, Shmueli K, Gil-Martinez AL, Leyland L-A, et al. Regional brain iron and gene expression provide insights into neurodegeneration in Parkinson’s disease. Brain. 2021;144:1787–98. 33704443 10.1093/brain/awab084 PMC8320305

[88] Lin J, Kang X, Xiong Y, Zhang D, Zong R, Yu X, et al. Convergent structural network and gene signatures for MRgFUS thalamotomy in patients with Parkinson’s disease. Neuroimage. 2021;243:118550. 34481084 10.1016/j.neuroimage.2021.118550

[89] Kedlian VR, Donertas HM, Thornton JM. The widespread increase in inter-individual variability of gene expression in the human brain with age. Aging. 2019;11:2253–80. 31003228 10.18632/aging.101912 PMC6520006

[90] Martins D, Giacomel A, Williams SCR, Turkheimer F, Dipasquale O, Veronese M. Imaging transcriptomics: Convergent cellular, transcriptomic, and molecular neuroimaging signatures in the healthy adult human brain. Cell Rep. 2021;37:110173. 34965413 10.1016/j.celrep.2021.110173

[91] Markello RD, Misic B. Comparing spatial null models for brain maps. Neuroimage. 2021;236:118052. 33857618 10.1016/j.neuroimage.2021.118052

[92] Alexander-Bloch AF, Shou H, Liu S, Satterthwaite TD, Glahn DC, Shinohara RT, et al. On testing for spatial correspondence between maps of human brain structure and function. Neuroimage. 2018;178:540–51. 29860082 10.1016/j.neuroimage.2018.05.070 PMC6095687

[93] Jack CR, Andrews SJ, Beach TG, Buracchio T, Dunn B, Graf A, et al. Revised criteria for the diagnosis and staging of Alzheimer’s disease. Nat Med. 2024;30:2121–4. 38942991 10.1038/s41591-024-02988-7 PMC11630478

[94] Ecker C, Pretzsch CM, Leyhausen J, Berg LM, Gurr C, Seelemeyer H, et al. Transcriptomic decoding of surface-based imaging phenotypes and its application to pharmacotranscriptomics. Nat Commun. 2025;16:6727. 40691132 10.1038/s41467-025-61927-3 PMC12279933

[95] Noori A, Mezlini AM, Hyman BT, Serrano-Pozo A, Das S. Systematic review and meta-analysis of human transcriptomics reveals neuroinflammation, deficient energy metabolism, and proteostasis failure across neurodegeneration. Neurobiol Dis. 2021;149:105225. 33347974 10.1016/j.nbd.2020.105225 PMC7856076

[96] Cappelletti C, Henriksen SP, Geut H, Rozemuller AJM, Van De Berg WDJ, Pihlstrøm L, et al. Transcriptomic profiling of Parkinson’s disease brains reveals disease stage specific gene expression changes. Acta Neuropathol. 2023;146:227–44. 37347276 10.1007/s00401-023-02597-7 PMC10329075

[97] Gao C, Zhou H, Liang W, Wen Z, Liao W, Xie Z, et al. Proteome-wide association study for finding druggable targets in progression and onset of Parkinson’s disease. CNS Neurosci Ther. 2025;31:e70294. 40008429 10.1111/cns.70294 PMC11862824

[98] Bustamam A, Sarwinda D, Ardenaswari G. Texture and gene expression analysis of the MRI brain in detection of Alzheimer’s disease. J Artif Intell Soft Comput Res. 2018;8(2):111–20.

[99] Askenazi M, Kavanagh T, Pires G, Ueberheide B, Wisniewski T, Drummond E. Compilation of reported protein changes in the brain in Alzheimer’s disease. Nat Commun. 2023;14:4466. 37491476 10.1038/s41467-023-40208-x PMC10368642

[100] Pérez-González AP, García-Kroepfly AL, Pérez-Fuentes KA, García-Reyes RI, Solis-Roldan FF, Alba-González JA, et al. The ROSMAP project: aging and neurodegenerative diseases through omic sciences. Front Neuroinform. 2024;18:1443865. 39351424 10.3389/fninf.2024.1443865 PMC11439699

[101] Biancalani T, Scalia G, Buffoni L, Avasthi R, Lu Z, Sanger A, et al. Deep learning and alignment of spatially resolved single-cell transcriptomes with tangram. Nat Methods. 2021;18:1352–62. 34711971 10.1038/s41592-021-01264-7 PMC8566243

[102] Zhang M, Pan X, Jung W, Halpern AR, Eichhorn SW, Lei Z, et al. Molecularly defined and spatially resolved cell atlas of the whole mouse brain. Nature. 2023;624:343–54. 38092912 10.1038/s41586-023-06808-9 PMC10719103

