# Supplementary material for: Application of the Allen Human Brain Atlas in Alzheimer’s disease and Parkinson’s disease
Source: Transl Neurodegener. 2026 Jul 23;15:33. doi: 10.1186/s40035-026-00566-0 (PMC13393942; doi:10.1186/s40035-026-00566-0)
Supplement: Supplementary file 1 — Additional file 1. Methods. [file 40035_2026_566_MOESM1_ESM.docx]

**Methods**

**Search strategy**

This study followed the guidelines of systematic reviews, the Preferred Reporting Items for Systematic Review and Meta-analysis guidelines (PRISMA) (**Table S2**)^[1]^. Four databases, MEDLINE, EMBASE, Web of Science, and Scopus, were searched for relevant studies till July 2025. The search strategy was as follows: (Alzheimer's disease OR Parkinson's disease OR neurodegenerative diseases OR dementia OR Alzheimer OR Parkinson OR neurodegenerat* OR dementia) AND (transcriptome OR RNA-seq OR microarray OR spatial transcriptomics OR imaging transcriptomic OR RNA sequencing OR gene expression atlas) AND (Brain OR Allen Human Brain Atlas). During the screening process, additional relevant articles were identified and included through citation tracking and literature network exploration.

**Selection criteria**

Studies were included if they met the following criteria: (1) investigated transcriptomic data from the Allen Human Brain Atlas (AHBA); (2) focused on Alzheimer’s Disease (AD) or Parkinson’s Disease (PD); (3) integrated or referenced transcriptomic data in neurobiological analyses. Our review encompasses a comprehensive spectrum of methodological approaches utilizing the AHBA. Specifically, we included studies employing: (1) direct spatial mapping of gene expression to imaging features; (2) cell-type deconvolution to estimate cellular composition; (3) gene set enrichment analysis to identify functional pathways; and (4) the construction of personalized computational models (e.g., whole-brain network simulations) informed by AHBA data. By covering these diverse strategies, we aim to provide a holistic overview of how the AHBA is currently leveraged in imaging transcriptomics. Studies were excluded if they: (1) were preprints without peer review, or conference abstracts without a full paper; (2) were published in languages other than English; (3) had no independent results of AD or PD.

**Data extraction and Quality assessment**

All records identified through the database search were first screened by title and abstract. Full texts of potentially eligible articles were subsequently reviewed against the predefined inclusion and exclusion criteria. For each included study, the following data were extracted: disease category, first author and year of publication, aim of study, main results, sample size, imaging modalities, AHBA data processing, sample assignment, data normalization, gene filtering, association methods between neuroimaging data and AHBA, testing of significance, biological interpretation, and code availability. The included studies were evaluated using the Joanna Briggs Institute Critical Appraisal Checklist to assess their quality and risk of bias^[2]^. Two researchers (Yi Xiao and Shichan Wang) independently performed study screening, data extraction, and quality assessment. Discrepancies were resolved through discussion and consultation with a senior reviewer (Yanbing Hou).

**Results**

**Search results and characteristics**

The initial database search yielded 14,402 records. After removal of duplicates (n = 5234) and exclusion of ineligible records based on title and abstract screening (n = 9168), 60 studies met the eligibility criteria and were included in the final analysis. The detailed study selection process is illustrated in **Figure 1.** Among the included studies, 39 focused on AD and 21 focused on PD. Detailed study characteristics and quality assessment scores are summarized in **Table S1** and **Table S2**, respectively.

[1] Liberati A, Altman D G, Tetzlaff J, et al. The PRISMA statement for reporting systematic reviews and meta-analyses of studies that evaluate healthcare interventions: explanation and elaboration[J]. Bmj, 2009, 339: b2700.

[2] Moola S, Tufanaru C, Aromataris E, et al.: Chapter 7: Systematic Reviews of Etiology and Risk, 2020.
