# Supplementary material for: Application of the Allen Human Brain Atlas in Alzheimer’s disease and Parkinson’s disease
Source: Transl Neurodegener. 2026 Jul 23;15:33. doi: 10.1186/s40035-026-00566-0 (PMC13393942; doi:10.1186/s40035-026-00566-0)
Supplement: Supplementary file 3 — Additional file 3. Table S2. Critical appraisal of papers included in the systematic review. [file 40035_2026_566_MOESM3_ESM.docx]

**Table S2. Critical appraisal of papers included in the systematic review**

| **Diseases** | **Authors** | **Year** | **Checklist criteria** | | | | | | | | |
| --- | --- | --- | --- | --- | --- | --- | --- | --- | --- | --- | --- |
|  |  |  | Q1 | Q2 | Q3 | Q4 | Q5 | Q6 | Q7 | Q8 | Total (%) |
| AD | Yang et al. | 2025 | 2 | 2 | 2 | 2 | 2 | 2 | 1 | 2 | 93.75% |
| AD | Chu et al. | 2025 | 2 | 2 | 2 | 2 | 1 | 1 | 1 | 2 | 81.25% |
| AD | Sepulcre et al. | 2018 | 2 | 2 | 1 | 2 | 2 | 1 | 1 | 1 | 75.00% |
| AD | Mattssonet al. | 2019 | 2 | 2 | 1 | 2 | 1 | 1 | 2 | 1 | 75.00% |
| AD | Yao et al. | 2024 | 2 | 2 | 2 | 2 | 1 | 1 | 1 | 2 | 81.25% |
| AD | Sanchez-Rodriguez et al. | 2024 | 2 | 2 | 1 | 2 | 2 | 1 | 1 | 1 | 75.00% |
| AD | Mullins et al. | 2022 | 2 | 2 | 1 | 2 | 1 | 1 | 1 | 1 | 68.75% |
| AD | Anand et al. | 2025 | 2 | 2 | 2 | 2 | 1 | 1 | 1 | 1 | 75.00% |
| AD | Yu et al. | 2024 | 2 | 2 | 2 | 2 | 2 | 2 | 1 | 2 | 93.75% |
| AD | Zheng et al. | 2024 | 2 | 2 | 1 | 2 | 2 | 2 | 2 | 2 | 93.75% |
| AD | Montal et al. | 2022 | 2 | 2 | 1 | 2 | 2 | 1 | 1 | 2 | 81.25% |
| AD | Luan et al. | 2024 | 2 | 2 | 1 | 2 | 2 | 1 | 1 | 2 | 81.25% |
| AD | Mullins et al. | 2017 | 1 | 1 | 1 | 1 | 1 | 1 | 1 | 1 | 50.00% |
| AD | Mullins et al. | 2017 | 2 | 2 | 1 | 1 | 1 | 1 | 1 | 1 | 62.50% |
| AD | Patel et al. | 2020 | 2 | 2 | 1 | 2 | 1 | 1 | 1 | 1 | 68.75% |
| AD | Nugent et al. | 2020 | 2 | 2 | 1 | 2 | 1 | 1 | 1 | 1 | 68.75% |
| AD | Zhang et al. | 2021 | 2 | 2 | 1 | 2 | 1 | 1 | 1 | 1 | 68.75% |
| AD | Brusini et al. | 2024 | 2 | 2 | 2 | 2 | 2 | 1 | 1 | 1 | 81.25% |
| AD | Peng et al. | 2025 | 2 | 2 | 2 | 2 | 2 | 2 | 1 | 2 | 93.75% |
| AD | Zheng et al. | 2025 | 2 | 2 | 1 | 2 | 2 | 1 | 1 | 1 | 75.00% |
| AD | Yu et al. | 2023 | 2 | 2 | 1 | 2 | 2 | 2 | 1 | 2 | 87.50% |
| AD | Zhao et al. | 2024 | 2 | 2 | 1 | 2 | 1 | 1 | 1 | 1 | 68.75% |
| AD | He et al. | 2022 | 2 | 2 | 2 | 2 | 1 | 1 | 1 | 1 | 75.00% |
| AD | Zheng et al. | 2024 | 2 | 2 | 1 | 2 | 1 | 1 | 1 | 1 | 68.75% |
| AD | Wang et al. | 2024 | 2 | 2 | 1 | 2 | 2 | 2 | 1 | 2 | 87.50% |
| AD | Xie et al. | 2021 | 2 | 2 | 2 | 2 | 1 | 1 | 2 | 2 | 87.50% |
| AD | Zheng et al. | 2024 | 2 | 2 | 1 | 2 | 2 | 1 | 1 | 1 | 75.00% |
| AD | Lu et al. | 2024 | 2 | 2 | 1 | 2 | 1 | 1 | 1 | 1 | 68.75% |
| AD | Tang et al. | 2024 | 2 | 2 | 2 | 2 | 2 | 2 | 1 | 2 | 93.75% |
| AD | Mahinrad et al. | 2018 | 1 | 2 | 2 | 2 | 1 | 1 | 2 | 1 | 75.00% |
| AD | Li et al. | 2025 | 2 | 2 | 2 | 2 | 2 | 1 | 1 | 1 | 81.25% |
| AD | Groot et al. | 2021 | 2 | 2 | 1 | 2 | 1 | 1 | 1 | 1 | 68.75% |
| AD | Adewale et al. | 2021 | 2 | 2 | 1 | 2 | 1 | 1 | 1 | 2 | 75.00% |
| AD | Yao et al. | 2025 | 2 | 2 | 1 | 2 | 1 | 1 | 2 | 1 | 75.00% |
| AD | Park et al. | 2012 | 2 | 2 | 1 | 2 | 1 | 1 | 1 | 1 | 68.75% |
| AD | Pak et al. | 2024 | 2 | 2 | 1 | 2 | 1 | 1 | 2 | 1 | 75.00% |
| AD | Bustamam et al. | 2017 | 2 | 2 | 1 | 2 | 0 | 0 | 2 | 1 | 62.50% |
| AD | Lancour et al. | 2020 | 1 | 2 | 1 | 2 | 1 | 1 | 2 | 2 | 75.00% |
| AD | Baik et al. | 2022 | 2 | 2 | 1 | 2 | 1 | 1 | 1 | 1 | 68.75% |
| PD | Keo et al. | 2020 | 2 | 2 | 2 | 2 | 2 | 1 | 1 | 2 | 87.50% |
| PD | Freeze et al. | 2019 | 2 | 2 | 1 | 2 | 2 | 1 | 2 | 2 | 87.50% |
| PD | Zheng et al. | 2019 | 2 | 2 | 1 | 2 | 2 | 1 | 1 | 2 | 81.25% |
| PD | Yan et al. | 2020 | 2 | 2 | 1 | 2 | 2 | 1 | 1 | 2 | 81.25% |
| PD | Thomas et al. | 2021 | 2 | 2 | 1 | 2 | 2 | 2 | 2 | 2 | 93.75% |
| PD | Wang et al. | 2024 | 2 | 2 | 2 | 2 | 2 | 2 | 1 | 2 | 93.75% |
| PD | Ji et al. | 2025 | 2 | 2 | 2 | 2 | 2 | 2 | 1 | 2 | 93.75% |
| PD | Freeze et al. | 2018 | 2 | 2 | 1 | 2 | 2 | 1 | 1 | 2 | 81.25% |
| PD | Yan et al. | 2025 | 2 | 2 | 1 | 2 | 1 | 1 | 1 | 2 | 75.00% |
| PD | Keo et al. | 2021 | 2 | 2 | 2 | 2 | 2 | 1 | 1 | 1 | 81.25% |
| PD | Keo et al. | 2021 | 2 | 2 | 2 | 2 | 2 | 1 | 1 | 1 | 81.25% |
| PD | Zang et al. | 2023 | 2 | 2 | 1 | 2 | 2 | 2 | 2 | 2 | 93.75% |
| PD | Bu et al. | 2025 | 2 | 2 | 2 | 2 | 2 | 1 | 1 | 2 | 87.50% |
| PD | Pisani et al. | 2023 | 2 | 2 | 1 | 2 | 1 | 1 | 2 | 1 | 75.00% |
| PD | Li et al. | 2025 | 2 | 2 | 1 | 2 | 2 | 2 | 1 | 2 | 87.50% |
| PD | Zarkali et al. | 2020 | 2 | 2 | 1 | 2 | 2 | 2 | 2 | 2 | 93.75% |
| PD | Zarkali et al. | 2020 | 2 | 2 | 2 | 2 | 2 | 1 | 1 | 2 | 87.50% |
| PD | Rittman et al. | 2016 | 2 | 2 | 1 | 2 | 1 | 1 | 1 | 1 | 68.75% |
| PD | Vo et al. | 2023 | 2 | 2 | 1 | 2 | 2 | 2 | 1 | 2 | 87.50% |
| PD | Lin et al. | 2021 | 2 | 2 | 2 | 2 | 1 | 1 | 1 | 2 | 75.00% |
| PD | Basaia et al. | 2022 | 2 | 2 | 1 | 2 | 2 | 2 | 2 | 2 | 93.75% |

Abbreviations：AD, Alzheimer’s Disease; PD, Parkinson’s Disease. Scores: 2=the study/manuscript fulfilled the criteria; 1=it was unclear if the study/manuscript fulfilled the criteria; 0=the manuscript/study did not fulfil the criteria. The total score (%) is the sum score across all criteria.
